# Distribution-free Bayesian analyses with the DFBA statistical package

**DOI:** 10.3758/s13428-025-02605-6

**Published:** 2025-02-19

**Authors:** Richard A. Chechile, Daniel H. Barch

**Affiliations:** Psychology Department, Tufts University, 490 Boston Av., Medford, MA 02155 USA

**Keywords:** Bayesian software, Distribution-free statistics, Nonparametric methods, Robust inference

## Abstract

Nonparametric (or distribution-free) statistics have been widely used in psychological research because behavioral data can be messy and inconsistent with the Gaussian model for measurement error. Distribution-free procedures only use categorical or rank information, so they avoid the problems of outliers and violations of distributional assumptions. Yet frequentist nonparametric procedures are still subject to the limitation of relative frequency theory, which stems from the founding assumption that population parameters cannot be represented by probability distributions. Bayesian statistical methods, by contrast, allow for prior and posterior probability distributions for population parameters, so they rigorously provide experimental scientists with a probability representation of the population parameters of interest. The Bayesian counterpart for a set of distribution-free statistical methods is a relatively recent development. This paper is a detailed discussion of the DFBA package of R functions, which is designed for doing distribution-free Bayesian analyses for the common nonparametric procedures. Included in the package are functions that enable the user to explore the relative power for computer-based data that can be sampled from nine different probability models. The distribution-free procedures have almost the same power as the *t* test when the data are normally distributed, but for eight other alternative probability models, the distribution-free Bayesian procedures have greater power than the frequentist *t*.

## Introduction

This article presents the DFBA (Distribution-free Bayesian analysis) R package, a suite of tools for performing Bayesian analyses that are free from parametric assumptions.^1^ Over the past decade, a number of Bayesian analogs to frequentist nonparametric statistical procedures have been developed (e.g., Chechile, 2018; Chechile, 2020a; Chechile, 2020b; Chechile and Barch, 2022). Distribution-free tests are extraordinarily versatile and useful in part because of the absence of assumptions about the unknown error. Relative to parametric tests, distribution-free tests are typically more robust because they avoid the excessive influence of a few outlier observations, and these nonparametric procedures can be statistically more powerful when underlying data structures are non-Gaussian (Chechile, 2020b). The Bayesian version of the nonparametric procedures has additional advantages over frequentist nonparametric tests by enabling researchers to make precise probability statements about both the null and alternative hypotheses. In total, there are 14 functions in the DFBA package, and this paper discusses each of these functions in the context of the behavioral sciences.

The DFBA package was designed with ease-of-use as a guiding principle. The package, along with its documentation, is especially crafted so that both seasoned data analysts and newcomers to Bayesian statistical inference can easily use the DFBA functions for implementing Bayesian analyses with a syntax that is similar to the R commands for familiar frequentist nonparametric tests, such as those in the base R *stats* package. For example, the Bayesian version of the Mann–Whitney *U* test for analyzing two independent groups, denoted as *E* and *C*, (under the assumption of low prior information) may take the simple form of **dfba_mann_whitney (E, C)**.

The paper also provides a historical and theoretical background underlying frequentist and Bayesian statistics. In this context, a case is made for the value of Bayesian distribution-free methods for experimental scientists. Overall, the main goals of the paper are: (1) to provide a brief overview of the advantages of the Bayesian approach to statistical inference, (2) to discuss the benefits of distribution-free analysis, (3) to review Bayesian distribution-free methods, and (4) to serve as a guide for using the functions of the DFBA package.

### Why do Bayesian analyses?

The short answer to the question is Bayesian statistical inference is a better match to the needs of scientists than is the orthodox relative-frequency approach. In the frequentist framework, only operations that can be repeated indefinitely can have a probability, so population parameters and hypotheses cannot have a probability because they do not have a relative frequency. The developers of orthodox relative-frequency were quite clear about this point. As noted frequentist von Mises (1957, p.11) stated:

The rational concept of probability, which is the only basis of probability calculus, applies only to problems in which either the same event repeats itself again and again or a great number of uniform elements are involved at the same time. Using the language of physics, we may say that in order to apply the theory of probability, we must have a practically unlimited sequence of uniform observations.

Historically, the Bayesian approach was the initial method of statistical inference (i.e., using information obtained from an experiment to estimate an unknown population parameter). This approach dates back to the independent work by Bayes (1764) and Laplace (1774). For example, the Laplace analysis to estimate the success rate for a potentially biased coin assumed a uniform prior distribution for the population binomial parameter, and computed, via Bayes theorem, a posterior distribution for the parameter. Laplace used the principle of insufficient reason to support a uniform prior distribution (i.e., no reason to prefer any one specific value for the population rate parameter from any other). The resulting point estimate, interval estimate, and probabilistic statements about the parameter are directly based on the properties of the posterior distribution. While Bayes theorem was universally accepted as a valid mathematical fact, some scholars objected to the application of Bayes theorem by letting the population parameter have a probability distribution; see Porter (1986) and Stigler (1986) for historical accounts about the 19th-century scholars who were critical of the Bayes/Laplace approach to statistical inference. These 19th-century scholars reasoned that the population parameter was surely a constant, so it seemed erroneous to treat the parameter as a random variable. This concern resulted in the development by Ellis (1842) of the relative frequency alternative to the Bayesian inference framework. Thus, the frequentist approach was a deliberate theoretical choice to avoid the initial Bayesian approach to statistical inference.

Other critics of the Bayes/Laplace approach bolstered the philosophical argument against Bayesian inference and its implications for the probabilistic representation of parameters with an additional technical concern about the effects of a nonlinear transformation of the prior distribution (Bing, 1879; Fisher, 1922). The issue of nonlinear transformations was also raised with Bertrand’s paradox as an objection against Laplace’s principle of insufficient reason (Bertrand, 1889). So, after the Fisher paper in 1922, the relative-frequency approach was the consensus model for statistical inference and the orthodox frequentist framework became – and in many applied fields remains – the dominant paradigm (Zabell, 1989).^2^ A century of statistical practice and education has reinforced this dominance by reducing the complex mathematics undergirding frequentist statistics to relatively simple algebraic equations and software solutions that scientists, who are experts in fields other than statistics, could use and apply.

Yet, subsequent mathematical developments, since the time of Fisher’s paper in 1922, have paved the way towards a more broad framework for probability that enabled states of the world, parameters, and hypotheses about parameters to have a probability representation (de Finetti, 1937; Kolmogorov, 1933; Ramsey, 1931; Shannon, 1948). These developments showed from an information-theory perspective that even unknown constants could validly have a probability representation in terms of our *knowledge* about the parameter. In Section “Which prior distribution?” the topic of encoding prior information is discussed in general with examples where there is low prior knowledge as well as for cases where there is an informed prior. Furthermore, Chechile (2023) recently resolved both the Bing–Fisher problem and the Bertrand paradox by showing that the previous analyses of these two technical issues failed to properly adjust the prior distribution to account for a mathematical property associated with the magnitude of the differential for a transformed variate. Thus, neither the Bertrand’s paradox nor the Bing–Fisher problem are valid arguments in themselves against the Bayesian approach, and there are straightforward ways to encode the researcher’s prior knowledge about the population parameters.

However, applied fields have been slow to embrace the Bayesian approach for a number of reasons. First, it was not clear that the Bayesian approach was philosophically valid because of the arguments raised by frequentists such as Bertrand’s paradox and the Bing–Fisher problem. However, as pointed out in the previous paragraph, these arguments have been rebutted. Second, Bayesian statistics, prior to the development and use of Markov chain Monte Carlo methods (Gelfand & Smith, 1990), was often mathematically complex and difficult to use. Even after these algorithms were developed, the Bayesian approach was initially still not easy to use and to understand by researchers that lacked special training in these methods. Currently, however, software packages such as JASP (JASP, 2024) and the BayesFactor (Morey & Rouder, 2022) have made the Bayesian approach much easier to use. Guides have been also provided to assist users who are new to Bayesian analyses (see, Etz et al., 2018). The DFBA package (Barch & Chechile, 2023), which is the focus of the current paper, is a further step in software development that is especially designed with many vignettes to aid beginners to Bayesian statistics.

While the above developments in Bayesian methods have made the approach more accessible, the question remains: why is Bayesian statistics a more suitable statistical method? The answer to this question is the Bayesian approach to point estimation, interval estimation, and hypothesis testing is rigorously grounded in standard probability theory – no ad hoc procedures are needed. In contrast, frequentist statistics involves a host of complex ideas that are easily confused and misinterpreted. For example, because in the relative frequency framework unknown population parameters are never allowed to have a probability distribution, frequentists cannot directly use probability theory for answering statistical questions about the parameters. Instead decision-making algorithms are advanced that are predicated on the notion of (infinitely) repeated samples. The frequentist approach deals with the properties of the decision-making algorithm. For example, a *t* statistic under the assumption of no treatment effect between two conditions has a distribution over repeated samples. The *p* value associated with the *t* test for *a specific experiment* is thus not a probability for the hypothesis being examined in the experiment since hypotheses themselves do not have a relative frequency. Yet it is commonplace that researchers mistakenly treat *p* values as probabilities for the study. This error is so widespread that the *American Statistical Association* has formed a commission of scholars to alert researchers about this error. The panel pointed out that a *p* value is not a probability for either the null or the alternative hypotheses (Wasserstein & Lazar, 2016). As statistician Robert Matthews stated about null hypothesis significance tests (NHST):

The key concepts of NHST- and, in particular, *p* values-cannot do what researchers ask of them. Despite the impression created by countless research papers, lecture courses, and textbooks, *p* values below 0.05 do not ‘prove’ the reality of anything. Nor, come to that, do *p* values above 0.05 disprove anything (Matthews, 2021, p. 16).

In addition to the *p* value misinterpretation, frequentist practitioners also often misinterpret the frequentist confidence interval (Hoekstra et al., 2014). The confidence interval is the frequentist interval estimate for the population parameter (Clopper & Pearson, 1934). For example, for the binomial rate parameter, the confidence interval (CI) given the observed sample data is the interval where an assumed null hypothesis $\phi =\phi _*$ would fail to reject the hypothesis at a given $\alpha$ level. All points within the CI would retain the null hypothesis, and all points outside the CI would reject the null hypothesis. Unfortunately, this technical definition of the CI is widely misunderstood by users (Hoekstra et al., 2014). First, the confidence interval is not a probability interval because if it were a probability interval, then the parameter would have a probability distribution, which is forbidden in frequentist theory. Second, a set of $1-\alpha$ confidence intervals does not have a coverage probability over repeated samples that is equal to $1-\alpha$. For example, Chechile (2020b) showed that the coverage probability depended on the value of the binomial rate parameter $\phi$. For some $\phi$ values the coverage proportion is less than $1-\alpha$ and for other $\phi$ values it was greater than $1-\alpha$. For a simple case where $n=4$ and where $\alpha$ was set to .5, the overall average coverage probability for the CI intervals was .7152. Chechile (2020b) also examined the coverage proportion for a Bayesian interval estimate when each Monte Carlo sample had a uniform prior and when 50% highest-density intervals were computed. The overall Bayesian coverage rate was .5004. So the Bayesian interval estimates do converge on average to the value of $1-\alpha$, but the frequentist Clopper–Pearson CI does not.^3^

Several theorists have postulated that the misinterpretations of frequentist methods and their results may be due to a mismatch between those methods and the typical goals of scientific inference (Chechile, 2020b; Wagenmakers, 2007). For example, the above common conceptual mistakes are examples of frequentist practitioners trying to interpret tests of hypotheses and interval estimates for parameters in terms of a probability value. While it is reasonable for researchers to want to make such claims, the frequentist framework does not allow a parameter or a hypothesis about a parameter to have a probability value because they do not have a relative frequency. Moreover, if the confidence interval were an interval with a probability value for the population parameter, then the primary objective of orthodox frequentist statistics would be violated.

To make a probabilistic statement about a hypothesis or a statement about the population parameter being within a range of numbers requires stipulating a prior probability for the event, and it requires a method for revising the probability based on experimental results. This approach is exactly the Bayesian method of statistical inference. Thus, the restriction that frequentists impose on probability, as articulated by the above quote from von Mises, to events that have a relative frequency is an arbitrary restriction on what can possess a probability that harkens back to a time before information theory and before the Kolmogorov axioms of probability. In Bayesian statistics, the distribution of belief about the population parameter before the experiment is the prior distribution or simply *the prior*, and the distribution for the population parameter after collecting the data is the posterior distribution or simply *the posterior*. The transformation of the prior to the posterior is based on Bayes theorem. Chechile (2020b) also argued that Bayesian statistical results are less susceptible to user misunderstanding because Bayesian interval estimates and Bayesian tests of hypotheses are in fact probability statements about the parameters.

Besides the fundamental difference between the frequentist approach and the Bayesian approach in the theoretical treatment of population parameters, there is another important difference in how these two systems of statistical inference use the likelihood function. In general, a likelihood is the probability of a data outcome given a specific stipulation for the population parameter (or parameters). Both statistical inference systems use likelihoods, but they use likelihoods differently. In the frequentist framework, the data are treated as a random variable. For this discussion, let us suppose that there is a single population parameter, which is denoted as $\phi$, and the outcome from a study is denoted as *x*. After the data are collected, the frequentist approach involves computing the likelihood of both the observed data *as well as the likelihoods for all the more extreme non-observed outcomes* under the assumption of a single assumed value of the population parameter. If we denote the likelihood $P(x\,|\,\phi )$ as the probability of finding *x* as an observed outcome given a population parameter $\phi$, then the frequentist approach is based on the summed likelihood $\sum _{x=x_{obs}}^{x_{max}}P(x|\phi )$, where $x_{obs}$ is the observed value and $x_{max}$ is the largest possible (but not observed) outcome value. Thus, after the data are collected, the frequentist approach treats the experimental outcome from the study as a random variable for a single, assumed $\phi$ value, and both the likelihood for the observed outcome as well as the likelihoods for all the more extreme *non-observed outcomes* are computed. For Bayesian inference, however, a different likelihood function is computed because after data are collected the only relevant likelihood in Bayes theorem is the likelihood for the observed data. Moreover, Bayesian analysts find the likelihood for the data for all possible values for the population parameter (i.e., $P(x_{obs}|\phi$), where $\phi$ is a random variable that ranges over all possible values of the population parameter. In essence, Bayes theorem is the normalization of the likelihoods, which are weighted by the prior probability for each possible $\phi$ value. The frequentist inclusion of the likelihoods of unobserved outcomes can result in paradoxical inferences where the *same data can be found to be either significant or not significant simply as a result of the stopping rules used in the collection of the data* ((Barnard et al., 1962; Berger & Wolpert, 1988; Chechile, 2020b; Lindley & Phillips, 1976). These paradoxes are not found with the corresponding Bayesian analysis.

Thus, from a Bayesian viewpoint, hypotheses about parameters can have probability values that are revised upon obtaining experimental data. In Bayesian hypothesis testing there is a posterior probability for both the null hypothesis and the alternative hypothesis. However, from a frequentist framework, only one hypothesis (i.e., the null hypothesis) is *assumed to be correct* in order to assess the likelihood of obtaining the observed data along with the likelihood of more extreme non-observed outcomes. If the frequentist sum likelihood based on the null hypothesis is less than a preset $\alpha$ value, then the user rejects the null hypothesis. The finding of a significant result *does not mean there is a probability value for the alternative hypothesis because in relative-frequency theory hypotheses never have a probability.* Moreover, the failure to reject the frequentist null hypothesis does not result in a decision in favor of the null because the null hypothesis was assumed in the first place. In contrast, with Bayesian statistics evidence can be built for either the null or the alternative hypothesis, and there are explicit posterior probabilities for both hypotheses.

### Why do distribution-free analyses?

The answer to the above question is that there is utility in doing statistical inference in a simple fashion that is not dependent on unrealistic assumptions about the data. Typically, parametric statistics, regardless of the method of statistical inference, assumes that a continuous measured quantity consists of a true score plus some random Gaussian error that has the same variance across treatment conditions. For example, both the frequentist analysis-of-variance ANOVA model (Kirk, 2013) and the Bayesian ANOVA model (Rouder et al., 2017) make the above parametric assumptions. There are a number of reasons to seek an alternative method of analysis that does not depend on making parametric assumptions about the underlying error. First, there might be block-by-treatment interaction effects. That is, there might be random measurement error that varies with each member in the population. Second, the random error might not be Gaussian. Third, there might be a mixture of participants with different effects of both treatment and error variance. Fourth, parametric models are typically not robust in the sense that a few outlier observations can have an exaggerated influence resulting in biased conclusions (Huber, 1977). For these reasons, it is advantageous to use a distribution-free nonparametric statistical assessment because it is a minimalistic analysis that typically only utilize either rank or categorical information. So, even if a researcher has done a parametric statistical analysis, it is judicious nonetheless for a careful scientist to also do a corresponding nonparametric analysis to see if the main results still hold up without the assumption of the parametric error model.

Nonparametric statistics was developed initially from a frequentist framework. In the behavioral sciences, there are a number of excellent textbooks on frequentist nonparametric or distribution-free statistics, (e.g., Hollander and Wolfe, 1999; Marascuilo and McSweeney, 1977; Siegel and Castellan, 1988). In a review of Bayesian statistics in 1972, it was noted that the topic of distribution-free statistical analyses was an area where the Bayesian approach was embarrassingly silent (Lindley, 1972). Subsequent to that assessment by Lindley, a number of Bayesian researchers developed a complex set of models that unfortunately have the label of *Bayesian nonparametric models* (e.g., Ferguson, 1973; Ghosh and Ramamoorthi, 2003; Müller et al., 2015). However, this label is misleading because the Bayesian nonparametric models developed by these researchers are not minimalistic analyses that are free of parametric assumptions. Instead, these models are complex and involve hyper-parameter spaces of many dimensions. Thus, a truly minimalistic Bayesian analysis that is free of the assumption of Gaussian error in each condition is a relatively recent development (e.g., Chechile, 2020b). Consequently, in this paper we are deliberately using the term of *Bayesian distribution-free analysis*, to avoid confusion with the term *Bayesian nonparametric modeling*. The Bayesian distribution-free analyses are direct counterparts to the frequentist nonparametric methods.

### Overview of the DFBA package and the paper

The DFBA package consists of fourteen functions. Table 1 is an organizational chart that groups these functions into four categories. Each DFBA function has a prefix of *dfba_*, so for example, the Bayesian sign test function has the instruction name of *dfba_sign_test()*.

**Table 1:** Overview of the DFBA functions

General tools	Categorical data	Interval or ranked data	Bivariate association
beta_descriptive	binomial	sign_test	bivariate_concordance
beta_bayes_factor	beta_contrast	median_test	gamma
sim_data	mcnemar	wilcoxon	
bayes_vs_t_power		mann_whitney	
power_curve			

In terms of the organization of this paper, Section 2 is devoted to discussing seven functions that are connected with the beta distribution in some fashion. These functions are listed below:

- dfba_beta_descriptive(); see subsection 2.1,
- dfba_binomial(); see subsection 2.2.3,
- dfba_beta_bayes_factor(); see subsection 2.3.1,
- dfba_beta_contrast(); see subsection 2.4.2,
- dfba_mcnemar(); see subsection 2.5.2,
- dfba_sign_test(); see subsection 2.6.1,
- dfba_median_test(); see subsection 2.6.2

Section 3 explores the *dfba_wilcoxon()* and the *dfba_mann_whitney()* functions. In Section 4 the *dfba_sim_data()*, *dfba_bayes_vs_t_power()* and the *dfba_power_curve()* functions are discussed. These functions can help users design a forthcoming experiment by simulating results from various probability models in order to estimate Bayesian and *t* power. In Section 5 the *dfba_bivariate_concordance()* and the *dfba_gamma()* functions are discussed. These R functions enable Bayesian distribution-free measures of bivariate association. Finally, there are concluding remarks about the overall package and its use in psychological sciences in Section 6.

## Beta-related functions

There are a number of frequentist nonparametric procedures for within-subjects and between-subjects designs that are based on either: (1) categorical data, or (2) interval-scale data that are used to form two response categories. For *repeated-measurements* studies with categorical data, the frequentist McNemar test is the traditional change-detection procedure, whereas the *sign test* is employed for interval scale measurements that are organized into a positive difference versus a negative difference. For *between-subjects or unrelated samples* studies, the frequentist $\chi ^{2}$ *test* is used for categorical data, and the *median test* is used for interval-scale data that are grouped into two categories. These statistical procedures are described in many texts of frequentist nonparametric methods, (e.g., Hollander and Wolfe, 1999; Marascuilo and McSweeney, 1977; Siegel and Castellan, 1988). The Bayesian counterparts to these procedures are described in Chechile (2020b). In each case, the posterior is either a beta distribution or a distribution of differences of beta variates.

### Beta introduction and the dfba_beta_descriptive function

The beta distribution is a continuous function of two non-negative shape parameters that are denoted as *a* and *b*. This distribution was initially used in the famous (Laplace, 1774) paper on the inverse probability problem. In general, the beta probability density for a continuous random variable *x* on the $[0,\,1]$ interval is

1 $$\begin{aligned} f(x)= \left\{ \begin{array}{cl} \frac{\Gamma (a+b)}{\Gamma (a)\Gamma (b)}x^{a-1}(1-x)^{b-1},\,\, & 0 \le x \le 1,\, a>0, b>0, \\ 0 & \text{ elsewhere }. \end{array} \right. \end{aligned}$$

**Fig. 1:**
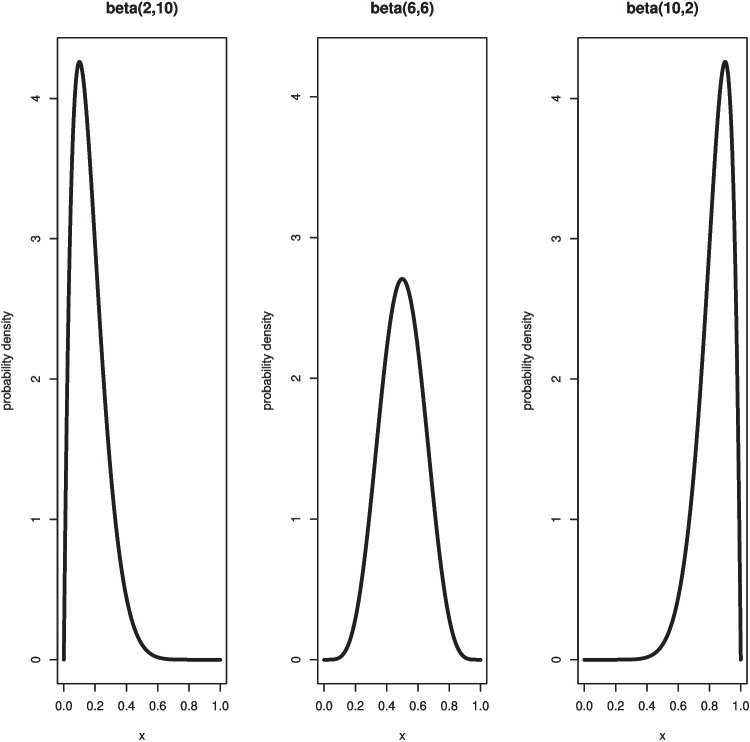
Three examples of beta distributions (beta (*a*, *b*)) probability densities for different values of the *a* and *b* shape parameters where $a+b=12$

The gamma function $\Gamma (y)$ in Eq. 1 is the generalization of the factorial to non-integer positive values. When *y* is an integer, $\Gamma (y+1)=y!$, but if *y* is not an integer, then $\Gamma (y+1)=y\Gamma (y)$ where $\Gamma (y)$ can be evaluated by the R command *gamma*. Since the *a* and *b* parameters are positive finite fixed values, the term $\frac{\Gamma (a+b)}{\Gamma (a)\Gamma (b)}$ is a constant, and this normalization constant assures that the cumulative probability over all values for *x* is 1. Figure 1 illustrates three examples of probability density displays for beta distributions with different values for the shape parameters where in each case $a+b=12$. When $a=b$ the beta density function is symmetric about the midpoint of .5. If $a>b$ then the distribution is negatively skewed, and if $a<b$ then the distribution is positively skewed. The uniform distribution on the $[0,\,1]$ interval is a beta distribution with $a=b=1$.

The R *stats* package provides functions for obtaining the *probability density*, *cumulative probability*, *quantiles*, and *random values* for a number of standard probability models. However, with distribution-free Bayesian analyses we are interested in additional properties of the beta distribution beyond those included in the basic R *stats* package. The *dfba_beta_descriptive()* function in the DFBA package provides additional descriptive information about a beta distribution. This function has three arguments, which are: (1) the beta *a* shape parameter, (2) the beta *b* shape parameter, and (3) the probability value for interval estimation; the names of these three arguments, respectively, are: *a*, *b*, and *prob_interval*. Note that this DFBA function uses the names of *a* and *b* for the arguments for the function rather than the names of *shape1*, and *shape2*, which are used for the base R beta functions for the *stats* package.

**Fig. 2:**
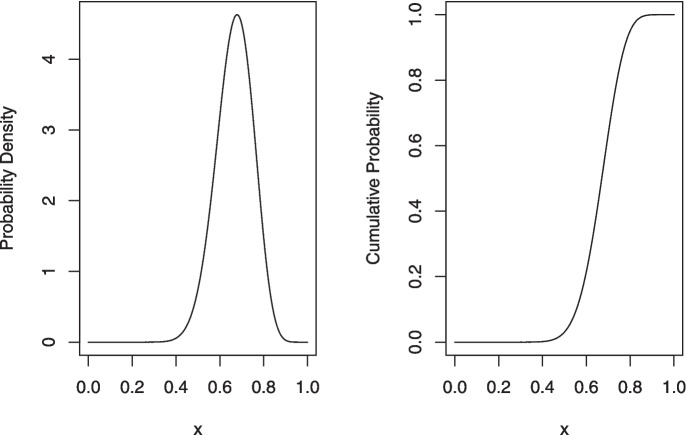
Example of the plot(dfba_beta_descriptive(a=20,b=10)) command

As the name implies, this function provides descriptive statistics for the beta distribution stipulated in the function call. The mean, median, and modal point estimates are provided. Furthermore, the variance of the distribution is computed. In addition, there are two interval estimates provided (i.e., an equal-tail interval and a HDI or *highest-density interval*). As an example, consider the following command: 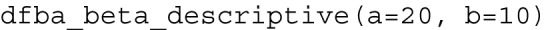 This command provides the following output: 
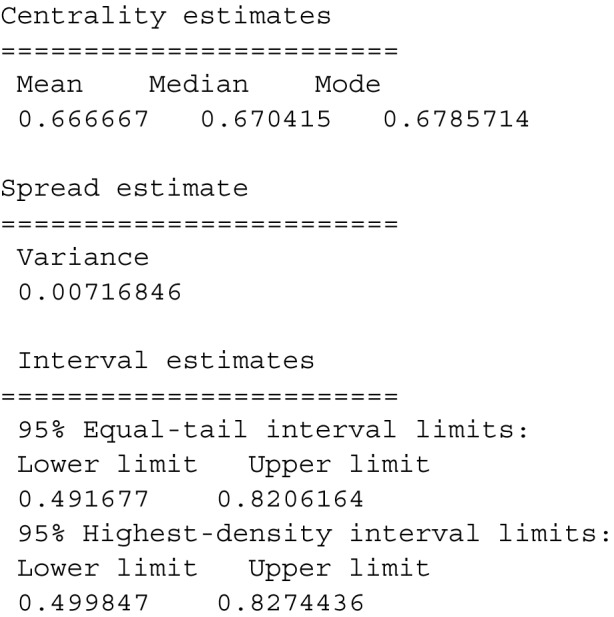


The *plot()* method produces plots of the beta distribution; an example of the syntax is: 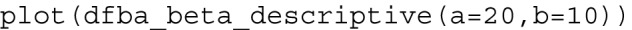


Figure 2 provides a probability density display along with a cumulative probability plot.

The cumulative probability at the value of *x* is the integration of the density function from 0 to *x*. For all *continuous* probability models, the probability for all points is zero because mathematical points are not a region or an interval. Yet there is a probability density for all points in the support domain; probability for an interval is the area of the probability density function over the interval.^4^

### The binomial function and Bernoulli processes

Before discussing the *dfba_binomial()* function it is useful to address several background theoretical points. In subsection “Bernoulli process likelihoods and the beta distribution”, the Bayesian approach is introduced.

#### Bernoulli process likelihoods and the beta distribution

Binary response data are common in psychological research; for example, an experiment in which each participant is tested to see if they can recall a previously presented target item. For another example, an investigator might want to see if a person can solve a puzzle (or not) in less than one minute. Studies of this type are examples of a Bernoulli process. All Bernoulli processes are cases where there are: (1) binary outcomes for each trial, (2) a common population parameter, which we will denote as the proportion $\phi$, for one of the two outcome categories, and (3) *independent* trials (i.e., the same $\phi$ parameter is valid for all trials). Researchers with this type of data have the statistical inference question associated with estimating the population $\phi$ parameter based on sample data of *n*1 observations for category 1 and *n*2 observations in category 2.

The likelihood function is the probability of a specific data outcome given a value for the population parameter. There are different Bernoulli likelihood functions in frequentist statistics depending on how the sampling was conducted. For Bernoulli processes, the likelihood is $L(n_1,n_2) = K_d \phi ^{n1}(1-\phi )^{n2}$ where $K_d$ is a number that differs with the stopping rules of the study. For *binomial* sampling the value of the number of trials *n* is fixed; whereas for *negative binomial* sampling (Haldane, 1945), the number of successes (or the number of failures) is fixed and the number of trials can vary. For binomial sampling, $K_d=\frac{n!}{n1!\,n2!}$. However for negative binomial sampling, the value of $K_d$ is different because there is a constraint that the last trial must be from a particular outcome category. For example, suppose for the negative binomial that it is stipulated that the number of successes must be *n*1 and that this condition is satisfied after *n*2 earlier failures, then $K_d=\frac{(n-1)!}{(n1-1)!n2!}$.

In frequentist statistics *the likelihood of the observed data is computed along with the likelihood of non-observed data that are more extreme*, so the value of $K_d$ for the Bernoulli process is important in the frequentist analysis because it is a component of each of the individual likelihood terms computed for the non-observed outcomes. However, in Bayesian statistics only the likelihood of the observed data are used because that is the only likelihood in Bayes theorem. Furthermore, in Bayesian statistics the term $K_d$ is not needed because it appears in both the numerator and the denominator of Bayes theorem, so it cancels out. Hence, in Bayesian statistics the likelihood function is generally only expressed as proportional to a function of the model parameters; that is for the Bernoulli processes it is $L \propto \phi ^{n1}(1-\phi )^{n2}$. This idea of only computing the likelihood of the observed data is called the *likelihood principle* (Barnard et al., 1962; Berger & Wolpert, 1988). Frequentist statistics violate the likelihood principle, and this practice can lead to the stopping-rule paradox where the same data can be either statistically significant or not significant in a frequentist test based on the stopping rule for the sampling (Chechile, 2020b; Lindley & Phillips, 1976). However, the same Bayesian likelihood function is used for *all Bernoulli processes* (e.g., both binomial sampling and negative binomial sampling), so the stopping-rule paradox does not occur.

It is well known that the posterior distribution for a Bernoulli process is another member of the beta family of distributions (Lindley & Phillips, 1976). That is, given the prior of $P(\phi )= \frac{\Gamma (a0+b0)}{\Gamma (a0)\Gamma (b0)}\phi ^{a0-1}(1-\phi )^{b0-1}$ and the likelihood of $L(n1,n2)=K_d \phi ^{n1}(1-\phi )^{n2}$, then the posterior distribution given the data of $D=(n1,\,n2)$ is

2 $$\begin{aligned} P(\phi \,|\,D)= &  \frac{\Gamma (a+b)}{\Gamma (a)\Gamma (b)}\phi ^{a-1}(1-\phi )^{b-1},\end{aligned}$$

3 $$\begin{aligned} a= &  a0+n1, \end{aligned}$$

4 $$\begin{aligned} b= &  b0+n2. \end{aligned}$$

A simple proof for this well-known fact can be found in Chechile (2020b). The fact that a beta prior for a Bernoulli process leads to a different posterior beta distribution is a special characteristic of the beta distribution; this property is called *Bayesian conjugacy*.

#### Which prior distribution?

For the Bayesian analysis of Bernoulli-process data, the prior distribution is taken to be a beta distribution with shape parameters of *a*0 and *b*0. The DFBA package allows users to employ whatever prior that they feel is suitable for the beta distribution provided that both *a*0 and *b*0 are positive numbers. While there is a default for the uniform prior of $a0=1$ and $b0=1$, users are free to input instead their preferred values for those arguments. However, which values for these shape parameters should the user endorse? This question has several possible answers. Users should choose the prior that represents the prior knowledge about the $\phi$ parameter that they want to base the statistical analysis upon. In general, there are two types of priors; one type is a low-information prior and the other type is an informative prior. Both types can be suitable. Let us discuss each type briefly.

*Low-information priors*

For a low-information prior, the maximally non-informative prior is the uniform distribution for Bernoulli processes, which is the case when $a0=b0=1$. This prior has maximum Shannon entropy (Chechile, 2020b). Another popular non-informative prior for Bernoulli processes is called the Jeffreys prior. Jeffreys (1946) proposed a non-informative prior in general that is proportional to the square root of the Fisher information matrix. Jeffreys developed this prior in response to the frequentist criticism (Bing, 1879; Fisher, 1922) that the uniform prior does not result in a uniform distribution when the researcher nonlinearly transforms the primary variate to an alternative variate. While the Jeffreys prior also changes with a nonlinear transformation, the resulting distribution is equal to the Jeffreys prior for the alternative nonlinear formulation. For this reason, there are a number of Bayesian statisticians who endorse the use of the Jeffreys prior (Bernardo & Smith, 1994; Zwickl & Holder, 2004). For Bernoulli sampling, the Jeffreys prior sets $a0=b0=\frac{1}{2}$. Chechile (2023) proposed an alternative response to the Bing–Fisher criticism. For this approach, a maximum entropy uniform prior is used for the primary $\phi$ variate, and a different prior that is proportional to the Jacobian of transformation is employed whenever the researcher adopts instead an alternative variate that is based on a nonlinear transformation of the $\phi$ variate. While the prior for the alternative variate is not a uniform distribution, the prior is nonetheless linked to the maximum entropy prior for the primary $\phi$ variate. So the original rationale of the Jeffreys prior is not a requirement. Furthermore, Chechile (2020b) showed that the uniform prior has maximum Shannon entropy (i.e., the least informative distribution) whereas the Jeffreys prior did not have maximum entropy.

While the posterior and prior are both beta distributions, the shape parameters are different for the uniform distribution and the Jeffreys prior. The prior has an effective sample-size weight of $a0+b0-2$, and the posterior has an effective sample-size weight equal to $a0+b0-2+n$. As an example, consider the uniform prior where $a0+b0-2$ (the prior sample-size weight) is 0, and the posterior sample-size weight is *n*. The Jeffreys prior, where $a0=b0=\frac{1}{2}$ has a prior sample-size weight equal to $-1$, and it has a posterior sample-size weight of $n-1$. Given a large enough collection of data, those two priors will have posterior distributions that have very similar sample-size weights because the data dominate the posterior distribution.

*Informative priors*

There are occasions when an informative prior is a good choice. For example, researchers may have good reasons for incorporating information that came from other studies. Whenever either *a*0 or *b*0 is greater than 1, it is effectively like having prior “observations” in the categories. So, to include data from past studies, the user can adjust the values for *a*0 and *b*0. This option is reasonable provided that the research protocols from previous studies are the same as the protocols for the new experiment. For example, it is possible for a carefully matched replication experiment that the prior *a*0 and *b*0 values are taken to be respectively the posterior *a* and *b* values from an earlier study. Bayesian statistics is a rigorous way to do a meta-analysis, provided that the measurement procedures are the same across the separate experiments.^5^ However, some investigators might deliberately choose to endorse a low-information prior even for a follow-up experiment because the researcher might want the analysis to be based on strictly the new data.

Another example where an informative prior is useful is when the data analyst wants to encode strong skepticism about the reality of an unusual phenomenon. Chechile (2020b) provided a hypothetical example of an experiment that was designed to see if a person could detect underground water with a divining (or dowsing) rod made from a Y-shaped branch cut from a tree. The hypothetical experiment consisted of a series of trials where on each trial the person, using a dowsing rod, had to detect which among six possible locations contained an underground drum filled with water rather than a drum filled with sand. To encode that the detection rate should be near a chance level of $\frac{1}{6}$, given a strong prior that there is no treatment effect, the prior was specified as a beta distribution with $a0=330$ and $b0=1650$, which is a prior with a mean at $\phi =\frac{1}{6}$. Effectively, the skeptical researcher has an opinion that is like assuming 329 prior successful detections and 1649 detection failures. So, the analyst encoded the skepticism about water divining as if there were 1978 previous trials. Despite this skeptical prior, if the person (the so called *water diviner*) could detect the underground drum that contained water on each of 36 separate test trials without any mistakes, then there would be a high posterior probability that the person, had a detection rate that was greater than $\frac{1}{6}$.

Suppose another analyst is not prejudiced against water divining, however this analyst still knows that there are five times as many drums without water than with water. Thus this nonprejudicial analyst might set $a0=\frac{1}{3}$ and $b0=\frac{5}{3}$. With this choice for the beta shape parameters, the effective prior sample size, which is $a0+b0-2$, is zero. However, unlike the uniform prior where *a*0 and *b*0 are 1 and where the prior sample size is also zero, the mean of the prior beta distribution for the nonprejudicial analyst is $\frac{1}{6}$ rather than $\frac{1}{2}$.

#### The dfba_binomial() function

The *dfba_binomial()* function is similar to the *dfba_beta_descriptive()* function in that it provides point and interval estimates for the posterior beta distribution given the specified prior and the observed values in the two categories of a Bernoulli process. The function has two required arguments, which are the values for the frequencies *n1* and *n2* in the two categories. The function also has three other optional arguments. These optional arguments are: *a0*, *b0*, and *prob_interval*. The shape parameters for the prior beta distribution are *a0* and *b0*, and these arguments have a default value of 1, which corresponds to the uniform distribution over the $[0,\,1]$ interval. The *prob_interval* argument is the probability value within the interval estimate; this argument has a default value of .95.

As an example, let us consider the Barthol and Ku (1953) study, which is discussed by Siegel and Castellan (1988). The participants were 18 students who learned two methods for tying a knot. Later, after a 4-h final exam, they were asked to tie the knot. There were 16 students who selected the first method and two students who selected the second method (i.e., the more recent method). Let $\phi$ be the population proportion for choosing to use the first methods after a stressful 4-h delay. Given a uniform prior for $\phi$ we can find the posterior point and interval estimates for $\phi$ by the following code: 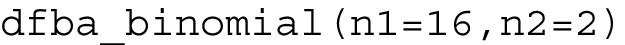


which resulted in the following output: 
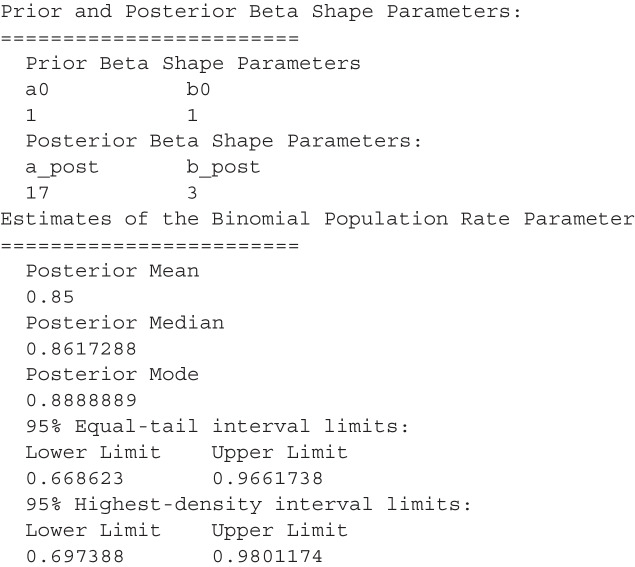


The plot() method produces a plot of the prior and posterior distribution; for this example, the call would be *plot(dfba_binomial(n1 = 16, n2 = 2))*.

The focus of the *dfba_binomial()* function is on the estimation of the $\phi$ parameter. For testing hypotheses about a posterior beta distribution, there is a separate DFBA function called *dfba_beta_bayes_factor()*. The next section describes how it is used for Bayesian tests about the Bernoulli rate parameter.

### The Bayes factor for a Bernoulli process

Unlike frequentist hypothesis testing, Bayesian statistics does not assume a specific value of the $\phi$ parameter. Instead, the critical assumption in the Bayesian analysis is the prior distribution for the parameter, which leads, upon the collection of data, to a posterior distribution. The posterior distribution contains all the information needed for statistical inference. So, if the user were interested in the hypotheses of $H_0:\, \phi \le .5$ and $H_1:\,\phi >.5$, then there are prior and posterior probabilities for each hypothesis.

The concept of the Bayes factor is widely used in Bayesian decision making. In general, the Bayes factor is a ratio of the likelihood of the data given two different models (Jeffreys, 1961). The models might be two rival probability models for the error (e.g., Gaussian error versus Weibull error). However, the models can also be two different hypotheses about a population parameter. It is this type of model comparison that is being tested with the DFBA package. The two hypotheses are generically denoted here as $H_1$ and $H_0$. The Bayes factor *BF*10 is the posterior odds ratio between $H_1$ and $H_0$ divided by the prior odds ratio between those two hypotheses (Jeffreys, 1961; Kass & Raftery, 1995).^6^ That is,

5 $$\begin{aligned} BF10 = \frac{P(H_1\,|\,D)\,/P(H_0\,|\,D)}{P(H_1)\,/P(H_0)}, \end{aligned}$$

where *D* denotes the data. Please note that the Bayes factor is relative to the prior odds ratio. For example, if the prior odds ratio is one-to-one and if the posterior odds ratio is 20-to-1, then the Bayes factor is 20. However, if the prior odds ratio is 4-to-1 while the posterior odds ratio is 20-to-1, then the Bayes factor is 5. Some critics of the Bayes factor (e.g., Aitkin, 1991; Grünwald, 2000; Lavine and Schervish, 1999; Liu and Aitkin, 2008) have stressed that this metric is dependent on the prior. These critics are correct, but in reality all Bayesian analyses are dependent on the prior, so this point is hardly a serious concern.

The Bayes factor *BF*10 can also be expressed as the ratio of the likelihoods for the data *D* between the two hypotheses. That is

6 $$\begin{aligned} BF10 = \frac{P(D\,|\,H_1)}{P(D\,|\,H_0)}. \end{aligned}$$

There is also the Bayes factor *BF*01, which is the posterior odds ratio of $H_0$ to $H_1$ divided by the prior odds ratio. In general, $BF01=\frac{1}{BF10}$. In Bayesian statistics, evidence can be built for either of the two hypotheses depending on the value for the larger of $(BF10,\,BF01)$.

There is more to the topic of Bayes factors beyond what is needed to discuss the computation of Bayes factors for a beta-distributed random variable (viz., Morey et al., 2016; Mulder and Wagenmakers, 2016; Schönbrodt and Wagenmakers, 2018; Stefan et al., 2019). A targeted tutorial about Bayes factors is provided for the reader in Appendix A. This brief tutorial provides a formal decision-theoretic argument for a three-alternative framework for Bayesian hypothesis testing along with some suggested guidelines for the use of Bayes factors. There is also an important discussion about the relative value of an interval-null hypothesis (e.g., $\phi \le .5$) versus a point-null hypotheses (e.g., $\phi =.5$). Furthermore, the appendix discusses some limitations and criticisms of Bayes factors.

#### The *dfba_beta_bayes_factor()* function

The function *dfba_beta_bayes_factor()* computes the Bayes factors *BF*10 and *BF*01 for user-specified beta distributions. The function can compute interval-type Bayes factors where the null and alternative hypotheses are mutually exclusive regions for the location of the population $\phi$ parameter. For example, the null region might be $\phi \le .5$ with the alternative hypothesis of $\phi >.5$. As another example, the null hypothesis might be a narrow interval centered at a chance level of .25, such as $H_0:.245 \le \phi \le .255$ where $H_1$ is the combination of the $0\le \phi <.245$ interval with the $.255 < \phi \le 1$ interval. The *dfba_beta_bayes_factor()* function can also compute the Bayes factor for a point-type null hypotheses such as $\phi =.5$, which has the corresponding alternative hypothesis of $\phi \ne .5$.

The *dfba_beta_bayes_factor()* function has four required arguments and two optional arguments. The shape parameters *a_post* and *b_post* for the posterior beta distribution are two of the required arguments. Another required argument is called *method*, which has two possible values, which are either the string “interval" or the string “point”; this argument stipulates the type of Bayes factor being computed. The last required argument is *H0*, which specifies the null hypothesis. If *method=“interval”*, then the *H0* argument must be the lower and upper limits of the null hypothesis (e.g., *H0=c(0,.5)*). If *method=“point”*, then the *H0* argument must be a single point (e.g., *H0=.5*). The function gives the user an error message if an absurd interval is stipulated, or if the argument to *H0* is inconsistent with the type of null hypothesis called for in the *method* specification. The two optional arguments are the beta shape parameters for the prior, *a0* and *b0*; each of these parameters has a default value of 1, which corresponds to a uniform prior.

As an example, suppose we wish to find the Bayes factor for the null hypothesis $H_0:\,\phi \le .5$ for the Barthol-Ku study discussed in subsection “The dfba_binomial() function

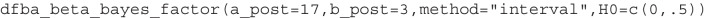



The output lists both BF10 and BF01 as well as the prior and posterior probabilities for both the null and alternative hypothesis. The BF10 value is listed as 2,743.963, so according to the guidelines discussed in Appendix A, the alternative hypothesis of $H_1:\,\phi >.5$ is “nearing certainty”. Nonetheless from the Bayesian framework, there still is a non-zero probability of .000364 for the null hypothesis.

There is no plot() method for the *dfba_beta_bayes_factor()* function. However, plots of the posterior and prior distributions for this study can be obtained from the *dfba_binomial()* function.

### Two or more independent beta variates

#### Theoretical context for the dfba_beta_contrast() function

In this subsection, we will focus on the case when there is a binary categorical response in each of *K* separate conditions. This case is a generalization from when there is a binary response measure in only one condition. When there are *K* groups, it is clear that the researcher is interested in comparing the effects of the conditions on the response rate. Each condition variate has a population proportion $\phi _i$, so the experimental design is implicitly addressing the question about how the population response rate parameters $\phi _i$, $i=1,\cdots ,K$ differ across conditions. The standard frequentist nonparametric procedure for addressing this question is to do a single $\chi ^{2}(df=K-1)$ test (Siegel & Castellan, 1988). This test assesses the point-null hypothesis that all the $\phi _i$ parameters are equal (i.e., $\phi _1=\phi _2=\cdots =\phi _K=\phi$). This type of hypothesis is commonly used in frequentist statistics, but it has been strongly criticized by Bayesian researchers. Even in the case when $K=2$, the difference in the rate parameters such that $\phi _1-\phi _2=0$ has a *probability measure of zero*. The difference has a non-zero *probability density*, but the *probability* that there is mathematical equivalence between the two parameters is zero. Chechile (2020b) argued that

Many disciplines have looked to statistics as a model for how to conduct research. Sciences do need to understand how to account for inferential uncertainty and errors. Unfortunately, the emphasis on sharp-null hypothesis testing has steered too many researchers using frequentist statistics towards wasting their time assessing hypotheses that are almost certain to be false. The sharp-null hypothesis is usually only retained because the sample size was too small. Instead, researchers should be asking more interesting scientific questions and should be using other tools. (p. 251)

Given that there is no doubt about the point-null hypothesis test in the limit of infinite sample size, then why design a statistical analysis in terms of this trivial statistical question? For this reason, the Bayesian approach is instead focused on estimating and evaluating a *contrast parameter* that compares how the population rate parameters differ from one another.

The *dfba_beta_contrast()* function enables the user to define and test a linear contrast parameter – denoted as $\Delta$ – that compares the $\phi _i$ parameters such that the contrast parameter is a value on the $[-1,\,1]$ interval. A contrast is defined by a vector of condition weights $(\psi _1,\,\psi _2,\,\cdots \,\psi _K)$ where the weights are real-value proportions such that the sum of all the positive weights is 1 and the sum of all the negative weights is $-1$; thus $\sum _{i=1}^{K}\psi _i=0$. For example, if there are four groups, the user might be interested in the contrast between the response rate in the first condition versus the average of the other three conditions. This contrast would correspond to the case where the coefficients or weights $\psi _i$, $i=1,\cdots ,4$, are $(-1,\,\frac{1}{3},\frac{1}{3},\frac{1}{3})$, which corresponds to $\Delta =-1\phi _1+\frac{1}{3}(\phi _2+\phi _3+\phi _4)$. The estimation of the $\Delta$ parameter and the evaluation of interval-null hypotheses about the $\Delta$ parameter are computed by the *dfba_beta_contrast()* function.

The Bayesian posterior distribution for each of the *K* independent conditions is a beta distribution with the shape parameters $a_{i}=a0_{i}+n1_{i}$ and $b_{i}=b0_{i}+n2_{i}$ where $n1_{i}$ and $n2_{i}$ are the response frequencies in the *i*th condition and where $a0_i$ and $b0_i$ are the corresponding beta shape parameters for the condition prior. For each condition the posterior mean and variance for $\phi _i$ are respectively $E(\phi _i)=\frac{a_i}{a_{i}+b_{i}}$ and $V(\phi _i)=\frac{a_i b_i}{(a_i+b_i)^{2}(a_i+b_i+1)}$. From elementary probability theory it is well known that a linear contrast of *K* independent random variates $X_i$ with coefficient weights of $\psi _i$, for $i=1,\cdots ,K$, have the respective mean and variance of $\sum _{i=1}^{K}\psi _{i}E(X_i)$ and $\sum _{i=1}^{K}\psi _{i}^{2}V(X_i)$. Thus, it follows that the expected value $E(\Delta )$ and variance $V(\Delta )$ of the posterior distribution for $\Delta$ are:

7 $$\begin{aligned} E(\Delta )= &  \sum _{i=1}^{K}\psi _i\,\frac{a_{i}}{a_{i}+b_{i}},\end{aligned}$$

8 $$\begin{aligned} V(\Delta )= &  \sum _{i=1}^{K}\psi _i^{2} \frac{a_i b_i}{(a_i +b_i)^{2}(a_i+b_i+1)}. \end{aligned}$$

Although the mean and variance for $\Delta$ are known via Eqs. 7 and 8, the distribution of $\Delta$, which is needed for interval estimates and tests of hypotheses, is not known. Nonetheless, we can approximate the distribution via random Monte Carlo sampling.

Random $\Delta$ values can be obtained by first drawing random values for each posterior $\phi _i$ for $i=1,\,\cdots ,\,K$. For example, for the *i*th condition, we can use the R *rbeta()* function to obtain *N* random values for $\phi _i$. Let us denote these *N* values as a vector $(\phi _{i1}, \,\phi _{i2},\,\cdots \,\phi _{iN})$. In total, there are *K* vectors of this sort. So, the element $\phi _{ij}$ is the *j*th random value from the *i*th posterior beta variate. There is a corresponding vector of $\Delta _j$ values where

9 $$\begin{aligned} \Delta _j = \psi _1 \phi _{1j}+\psi _2 \phi _{2j} +\cdots +\psi _K \phi _{Kj}, \end{aligned}$$

for $j= 1, \cdots , N$. The posterior probability that $\Delta >0$ is estimated by the proportion of the *N* random $\Delta _j$ values that are positive. Similarly, the quantiles for the contrast are estimated from the quantiles of the $\Delta _j$ values.^7^ 

#### The dfba_beta_contrast() function

The *dfba_beta_contrast()* function has three required arguments and four other optional arguments. The observed *n*1 frequencies for each condition are encoded into an argument vector that is called *n1_vec*, whereas the corresponding frequencies *n*2 are encoded into a vector argument called *n2_vec*. The third required argument is a vector of the contrast coefficients; this argument vector is called *contrast_vec*. The *K* elements of the contrast argument vector sum to 0, and the sum of the positive coefficients add to 1 while the sum of the negative coefficients add to $-1$. If the coefficients defined by the user are inconsistent with the above constraints, then an error message is generated.

The four optional arguments are called *a0_vec*, *b0_vec*, *prob_interval*, and *samples*. The *a0_vec* and *b0_vec* arguments are the prior beta distribution shape parameters. The default value for each parameter for each of the *K* separate conditions is 1, which corresponds to a uniform distribution as the prior. The *prob_interval* argument is the value for the interval estimate of the contrast $\Delta$ parameter, and it has a default of .95. Finally, the *samples* argument is the number of Monte Carlo random values that are sampled from each of the *K* conditions. The default for this argument is 10000.

The *dfba_beta_contrast()* function is an example of a subset of the DFBA programs that employ conventional Monte Carlo sampling. For each of the functions in this subset, there is an optional argument called *samples* that stipulates the number of random samples drawn. Statistical results from Monte Carlo simulations depend on a starting seed value for the pseudorandom numbers that are generated and also depend on the value for the *samples* argument. Later in this subsection, an example is provided that illustrates how the user can alter the random seed and the value for the *samples* argument to find limits on a key statistical result. While limits of a Monte Carlo simulation can be estimated with the current version of the DFBA package, we plan to assist the user in future revisions of the package by supplying a function-computed estimate for the range of the statistical results obtained via Monte Carlo sampling.

Let us illustrate the use of the *dfba_beta_contrast()* function by way of an analysis of a hypothetical study where the researcher examines four conditions and where the response measure is binary with eight trials per condition. The *n*1 frequencies in the four respective conditions are $1,\,4,\,6,\,7$ and the corresponding *n*2 frequencies are $7,\,4,\,2,\,1$. One contrast of interest is the comparison of group 1 versus the average of the other three groups. This contrast can be examined via the following commands:

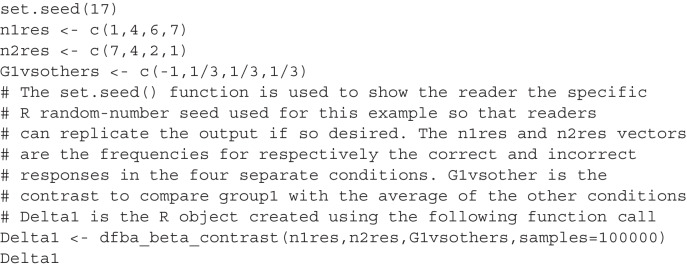



Note that the above code increased the value for the *samples* argument by a factor of ten from the default setting. The following output is generated.

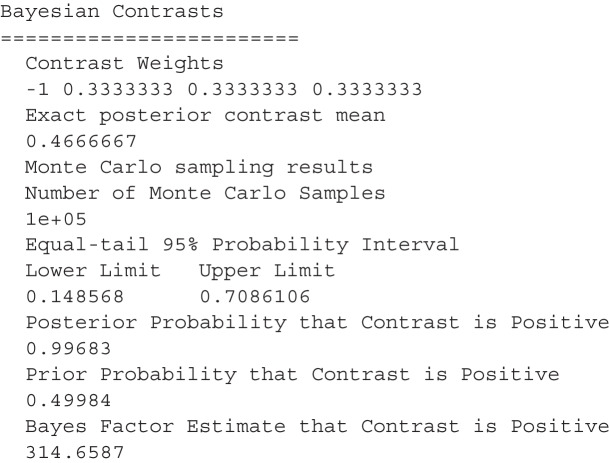



**Fig. 3:**
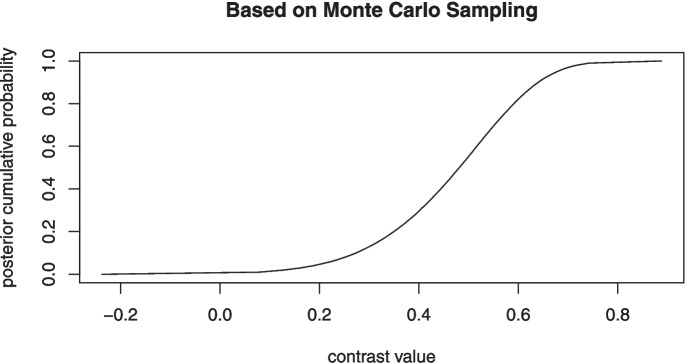
Example of the plot method associated with the dfba_beta_contrast() function

Given the large Bayes factor for the contrast, it is a good bet (as described in the Bayes factor tutorial in Appendix A) that the population value for $\phi _1$ is less than the average of the other $\phi _i$ parameters. The posterior cumulative distribution for the $\Delta$ parameter is obtained from the instruction *plot(Delta1)*, which is displayed in Fig. 3.

Given how the above contrast is defined, it is clear that the positive contrast indicates that the first condition is likely to have a lower response rate $\phi$ than the average of the other three conditions. Yet, the reader should be aware that the above value for the posterior probability of a positive contrast and the associated Bayes factor are the results from a Monte Carlo simulation. Hence, a subsequent rerunning of the above commands with a different random seed value is likely to yield different specific values for these quantities. For instance, a second execution of the same commands with a different seed of 45 yielded a posterior probability of .99733 rather than the above value of .99683. These two values are within .0005, which is a trivial difference. However, the user can gain even greater precision for the statistical estimates by increasing the value for the *samples* argument to an even larger number. For example, if the user sets the *samples* to one million, then the posterior probability is $.997132 \pm .00005$.

Other contrasts among the four conditions in this example are also possible. Two other contrasts of potential interest might be: (1) to compare condition 2 versus the average of conditions 3 and 4, and (2) to compare condition 3 versus 4. The contrast coefficient for these two contrasts are respectively *contrast_vec=c(0,-1,.5,.5)* and *contrast_vec=c(0,0,-1,1)*. This set of three contrasts is orthogonal because the dot product of the contrast coefficients between any two of the three contrasts is 0.^8^ Similar analyses of the other two contrasts did not result in a highly credible contrast because the 95% intervals included 0 and the Bayes factors were less than 19 (see Appendix A for suggested guidelines for interpreting the Bayes factor). That is, the 95% interval for the comparison between condition 2 and the average of conditions 3 and 4 is $(-.097,.5870)$, and the corresponding interval for the comparison between conditions 3 and 4 is $(-.265,.461)$. Also, the Bayes factors for these two contrasts were respectively 11.23 and 2.47.

### Bayesian McNemar test

#### Background for McNemar test

For the frequentist McNemar test (e.g., Siegel and Castellan, 1988) there are two within-block conditions or trials where the variate is binary. As an example, consider the data from Chechile (2020b, p. 217) where each of 72 participants were tested on consecutive trials. The stimuli for both trials were a triad of words from a common semantic category (e.g., flowers, spices, fruits, vehicles). The same semantic category was used on consecutive trials. If the participants correctly recalled all three items of a triad, then it was scored as a correct response. In the presence of the memory phenomenon known as *proactive interference*, performance on the second trial should be reduced for the participants who remembered correctly the triad from the first trial. The frequency data for this study are arranged in the following $2\times 2$ contingency table.

**Table:** 

	Correct on Trial 2	Incorrect on Trial 2	
Correct on Trial 1	6	21	27
Incorrect on Trial 1	2	43	45
	8	64	72

The frequentist McNemar procedure is a $\chi ^{2}$ statistical test that assesses the null hypothesis that there is no difference in the direction of change of the response rate between the two tests for the subset of people whose accuracy *differed* on the two trials. The frequentist McNemar test thus disregards the *consistent* cases (i.e., the (row 1, column 1) cell and the (row 2, column 2) cell). The McNemar procedure instead focuses on the *change* cases (i.e., the response rate for the (row 1, column 2) cell versus the response rate for the (row 2, column 1) cell). For the above data, this frequentist test would reject the sharp null, $\chi ^{2}(df=1)=15.7$, $p<.0007$. Note that the frequentist McNemar procedure is not designed to be a statistical test of independence, but rather it is a test of the symmetry of the changes in the binary response rate between the two trials. The Bayesian version of the McNemar test also focuses on the two cells where the participants are not responding the same (i.e., cells $n_{12}$ and $n_{21}$). Thus, only 23 participants are the basis of the Bayesian McNemar test for the above experiment, and 21 of these participants did in fact have an incorrect response if they remembered the trial 1 triad.

Chechile (2020b) pointed out that the Bayesian version of the McNemar procedure is straightforward since the subset *of the change cases* is a conditional Bernoulli process. In the Bayesian analysis, the population response-switching rate is called $\phi _{rb}$ where the *rb* subscript on the $\phi$ parameter denotes a randomized-block, which is a term that encompasses repeated-measures and paired-samples designs. Unlike the frequentist McNemar test, the Bayesian analysis does not assume the null hypothesis that the switching proportion is .5. Rather, there is a prior distribution assumed for the $\phi _{rb}$ parameter. The prior is a beta distribution because it is the result of a censored-Bernoulli process where the no-change cases are ignored and because the Bayesian analysis of all Bernoulli processes have a prior and posterior beta distribution (Lindley & Phillips, 1976).

**Fig. 4:**
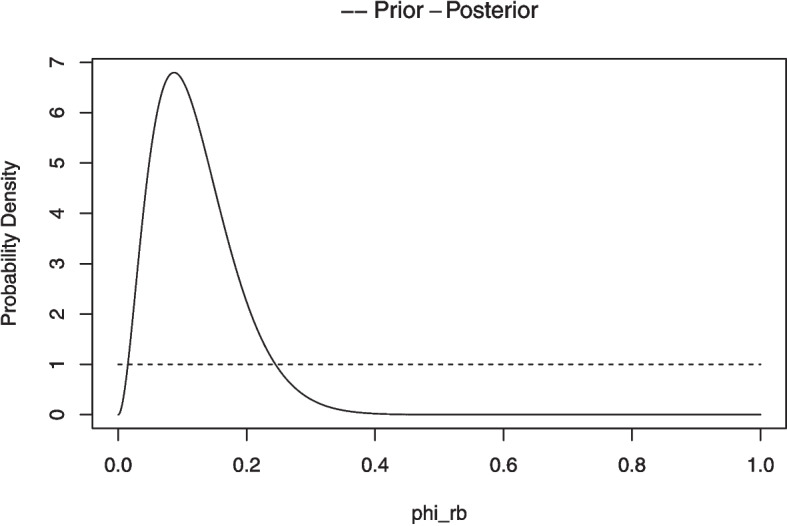
Example of the plot associated with the *dfba_mcnemar()* function

#### The *dfba_mcnemar()* function

The *dfba_mcnemar()* function has two required arguments that are the two observed frequencies for switching. One frequency is *n_01*, which is the number of switches from a 0 score on the pre-test to a 1 score on the post-test, and the other frequency is *n_10*, which is the number of switches from 1 to 0. There are also three other optional arguments; these arguments are *a0*, *b0*, and *prob_interval*, which have the same meaning as for the other DFBA functions with those names that have been discussed previously (see the *dfba_binomial()* function in Section “The dfba_binomial() function”). As an example, consider the following R instruction that creates a new R object, which is defined as *A*, and executes the Bayesian McNemar test for the data from the above proactive interference study: 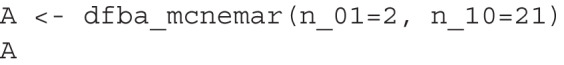


The output from this set of instructions is shown below.

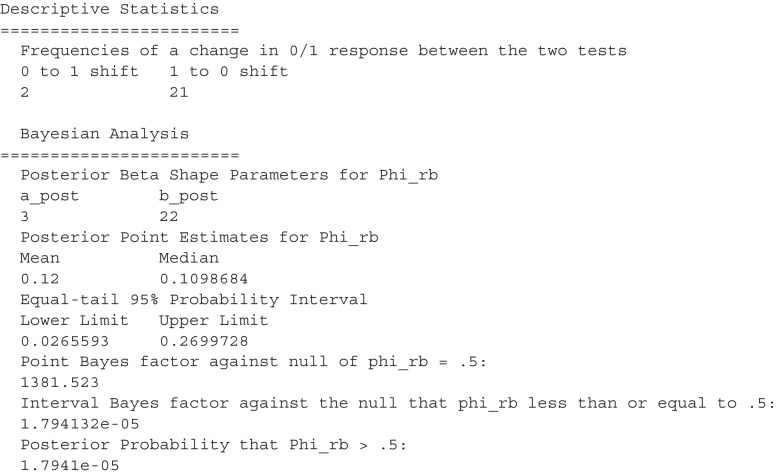



The $\phi _{rb}$ parameter is defined to be associated with an *improvement* between the first and second trial. So, the finding of $\phi _{rb}<.5$ would indicate that there was a decrease in the memory retention for the second trial. Since the posterior probability for $\phi _{rb}>.5$ is .00001794, it follows that the posterior probability for $\phi _{rb}<.5$ is .99998206. The experiment thus supports the hypothesis that a robust proactive interference effect occurred.

The instruction *plot(A)* provides plots of the prior and posterior density function for $\phi _{rb}$ parameter (see Fig. 4).

If the user wishes to suppress the plotting of the prior for any DFBA function that has a plot method, then add the optional argument *plot.prior = FALSE* to the plot function call.

### Bayesian sign and median tests

This subsection deals with two DFBA functions where the data are continuous response measures, but where the nonparametric analysis is based on the extraction of two discrete categories. For the sign test, the two variates are paired (i.e., it is a randomized-block design), and the binary classification is based on the difference between the conditions being either positive or negative. For the median test, the two variates are the scores from two *independent* conditions or groups, and the binary classification is based on a score being either above the combined median or not being above the combined median.

#### Bayesian sign test

The sign test deals with within-subjects (or within-block) data where there are continuous measures *Y*1 and *Y*2 for the two conditions. For both the frequentist and the Bayesian sign test, a difference score $d=Y1-Y2$ is obtained between the two measures per block. If the *d* score is zero, then that block is ignored. The non-zero *d* scores are then categorized as either positive or negative. The statistical inference is about the population proportion of positive differences, which we can denote as $\phi$. The frequentist test assumes the null hypothesis that $\phi =.5$ (Siegel & Castellan, 1988); whereas the Bayesian sign test (Chechile, 2020b) assumes a prior distribution for $\phi$.

The *dfba_sign_test()* function has two required arguments and three optional arguments. The two required arguments are the responses in the two respective conditions, and these arguments are called *Y1* and *Y2*. These arguments are separate paired vectors. That is, the *Y*1[*i*] value needs to be the score for the *i*th block in the *Y*1 condition, whereas the score in the same block in the *Y*2 condition is *Y*2[*i*]. The three optional arguments are: *a0*, *b0*, and *prob_interval*. These arguments have the same meaning as the other DFBA functions with those same argument names (e.g., see the *dfba_binomial()* function in Section “The dfba_binomial() function”).

The following R code illustrates the use of the *dfba_sign_test()* function for a hypothetical set of paired values.

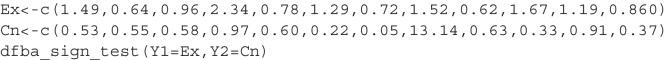



This code yields the following output:

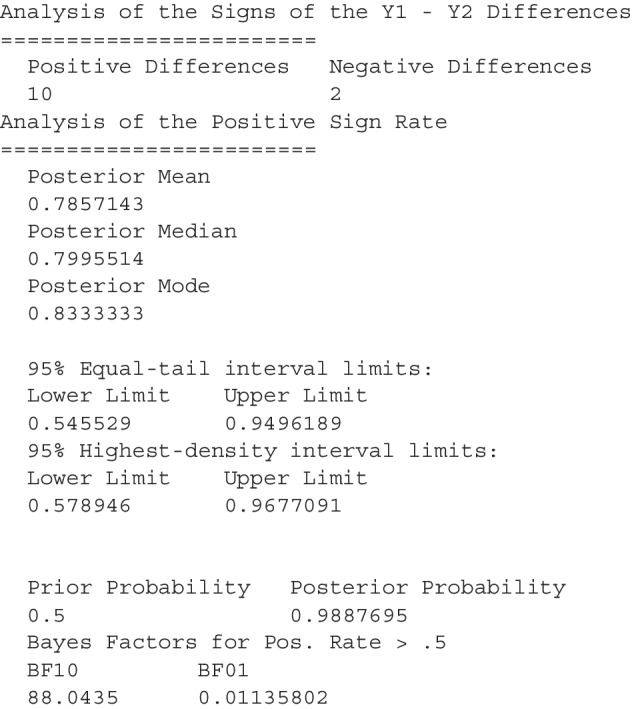



These results strongly support the hypothesis $H_1:\,\phi >.5$. The data in this example can also be examined with the frequentist parametric *t* test. It would be expected that the *t* would also detect that there is a difference between the two within-block conditions. The following R function would implement the frequentist *t* test for the data in this example: 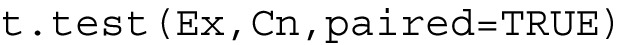


Please note that this command surprisingly fails to reject the null hypothesis on the parametric *t* test ($t=-.3887,\,p=.7049$). This example illustrates that on some occasions a nonparametric test is more sensitive for detecting a condition difference than the corresponding parametric test. The reason for this result is that the eighth score for the *Y2* measure is an outlier that has a large influence on the parametric *t* test. The outlier decreases the mean difference between the conditions, and it increases the standard error. Yet for the Bayesian sign test, the outlier does not overly influence the statistical analysis. The example thus underscores the value of routinely doing a simple nonparametric test even if a parametric procedure was initially employed.^9^ Often a distribution-free statistical test will confirm a strong effect, and on occasion it will detect an effect that was missed with the parametric procedure. In subsection “The dfba_bayes_vs_t_power() function” there is a more extensive discussion of the power of some of the distribution-free procedures relative to the parametric *t* test.

#### Bayesian median test

Siegel and Castellan (1988) discuss the frequentist median test to assess the point-null hypothesis that the proportion of responses above the combined median is the same for each of two independent (or between-groups) conditions. Chechile (2020b) provided an alternative Bayesian analysis for this procedure where the sample sizes in the two independent groups can be unequal. The Bayesian approach (like the frequentist approach), first computes the combined median of all the observed scores, and based on this value a $2 \times 2$ table of frequencies is generated. The rows are the frequencies of the scores that are above the combined median versus the frequencies of the scores that are either at or below the combined median. The columns are the frequencies of the scores that belong to each independent group, which are denoted as either *E* for *Experimental* or *C* for *Control*. The names *Experimental* and *Control* are simply generic labels for the two independent conditions. The frequentist Median test is a chi-square statistical test to assess the assumed point-null hypothesis of no differences between the two conditions (Siegel & Castellan, 1988).

The Bayesian analysis of the median-test procedure focuses on examining the above-median responses to estimate the population proportion of the above-median responses that are in the *E* group. Because the outcomes are binary, the prior and posterior distributions for this parameter are beta distributions. Thus, the Bayesian analysis, unlike the frequentist analysis, does not assess an assumed point-null hypothesis. Instead, the *dfba_median_test()* function assesses the null hypothesis that $H_0:\,\phi \le \frac{nE}{nE+nC}$ where *nE* and *nC* are the frequencies in the respective *E* and *C* groups. The null hypothesis is the hypothesis that the population proportion above the sample combined median from the *E* group is less than or equal to the sample base rate for the *E* group; whereas the alternative hypothesis for this null is $H_1:\,\phi >\frac{nE}{nE+nC}$.

The *dfba_median_test()* function has two required arguments, which are the *E* and *C* vectors of values. The function also has two optional arguments, which are the *a0* and *b0* shape parameters for the prior distribution that have the default value of 1. The following code illustrates the use of the function:

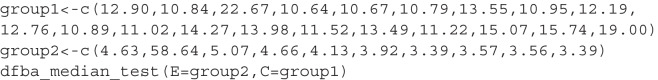



The above code result in the following output:

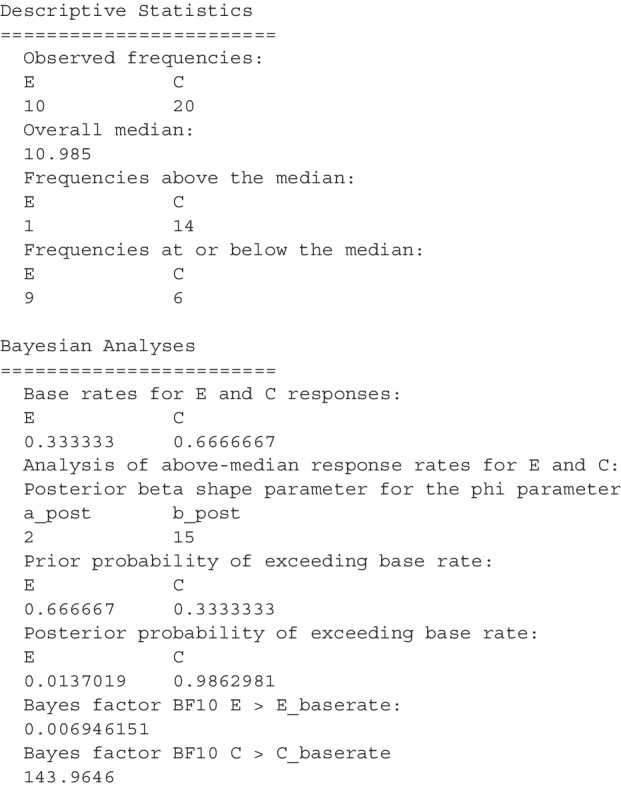



Please note that there are twice as many observations in the C condition than in the E condition, so the base rate for a C condition response for the above-median set of observations is $\frac{2}{3}$. Yet all but 1 of the 15 above-median values were from the C condition, so the C group is over performing compared to its expected base rate. The Bayesian analysis results in a high posterior probability of .986 that an above-median response from the C condition exceeds its expected base rate. Because the $\phi$ parameter is the population proportion of E condition responses that are above its expected base rate, the posterior probability for $\phi$ has beta shape parameters of *a_post* and *b_post*, which are respectively 2 and 15 as shown in the above output. Thus, the response rate for the E group among the above-median category is *below its expected base rate*. The *dfba_median_test()* function does not have a plot function; however, the *dfba_beta_descriptive()* function can be used to see the posterior density function and the cumulative distribution for the $\phi$ parameter (see subsection “Beta introduction and the dfba_beta_descriptive function”).

**Table 2:** An example of the Wilcoxon signed-rank procedure

*i*	$Y_1$	$Y_2$	$d_i$	Rank($|d_i|$)	Signed Rank
1	77.9	77.2	0.7	1	1
2	89.2	66.0	23.2	9	9
3	78.2	69.2	9.0	5	5
4	50.0	58.2	–8.2	4	–4
5	94.5	74.0	20.5	8	8
6	73.1	88.3	–15.2	7	–7
7	77.5	77.5	0	-	-
8	70.6	74.6	–4.0	2	–2
9	89.4	78.4	11.0	6	6
10	77.6	72.2	5.4	3	3

## DFBA alternatives to the *t* test

There are two classic nonparametric methods that are the counterpart to the frequentist *t* test. They are distribution-free methods because they only use the rank-order information of the continuous response measures. These tests are the Wilcoxon and the Mann–Whitney procedures. The Wilcoxon method is used when the scores from the two conditions are matched or paired, such as for a within-subjects experiment. The Mann–Whitney procedure is used when the two conditions are independent conditions, such as for a between-subjects experiment.

### Bayesian Wilcoxon analysis

The frequentist Wilcoxon procedure is the nonparametric counterpart to the matched-*t* test. The sample statistics are $T^{+}$ and $T^{-}$, although these two statistics are not independent, so effectively only one statistic is needed. It is standard to use the $T^{+}$ measure. To see how this statistic is computed, let us consider the set of ten continuous matched measurements shown in Table 2, which are denoted as $Y_1$ and $Y_2$.

While the matched-*t* test is based on the difference score values $d_i$ where $d_i=Y_1(i)-Y_2(i)$, the Wilcoxon procedure instead ranks the absolute values of the $d_i$ provided that the difference is not zero. For this example, in block $i=7$, the difference is zero, so this block is ignored; the deleting of blocks where $d=0$ is the standard practice for the frequentist Wilcoxon procedure (Siegel and Castellan (1988)). Consequently, for the Wilcoxon procedure, there are effectively only $n=9$ blocks for the above example rather than ten. The rank value for the maximum absolute value is *n*, whereas the lowest absolute $d_i$ value is ranked 1. In the next step, the ranks are converted to signed ranks (i.e., the sign of the $d_i$ is attached to the rank of $|d_i|$). The $T^{+}$ statistic is the sum of the positive ranks, which for the present example is equal to the value of 32. The $T^{-}$ statistic is the sum of the ranks that have a negative sign; however, $T^{-}$ is also a positive value. For this example, $T^{-}$ is 13. In general, $T^{+}+T^{-}=\frac{n(n+1)}{2}$. In this example, $n=9$, so $T^{+}+T^{-}=\frac{9(10)}{2}=45$. The frequentist Wilcoxon test is based on the distribution of the $T^{+}$ statistic when the sign-bias parameter is assumed to be the sharp-null hypothesis value of .5. However, for the Chechile (2018) Bayesian analysis, the sample value for $T^{+}$ for *n* blocks is treated as a fixed quantity upon the completion of data collection, and the population value for the Wilcoxon sign-bias parameter $\phi _w$ is considered to be the unknown parameter that is being estimated.

When the number of blocks *n* is small (i.e., $n\le 24$), the posterior distribution for $\phi _w$ is approximated in the Chechile (2018) approach by a discrete probability distribution. For the discrete approximation, 200 candidate values for $\phi _w$ that range from .0025 to .9975 in steps of .005 are examined.^10^ For each candidate value $\phi _{wi}$, a large number of Monte Carlo samples are drawn based on that candidate value. That is, the integers from 1 to *n* are randomly assigned with a sign value where $\phi _{wi}$ is the probability for a positive sign. This process results in a sample $T^{+}$ value.^11^ For a given $\phi _{wi}$, the proportion of the Monte Carlo samples where the sampled $T^{+}$ value equals the observed $T^{+}$ is an approximation of the likelihood $p(T^{+}\,|\,\phi _{wi})$. This likelihood is multiplied by the prior for $\phi _{wi}$, and this product results in one of the 200 numerators for Bayes theorem. The process is repeated for each of the 200 values for $\phi _{wi}$. The denominator of Bayes theorem is the sum of the 200 possible numerator values. Bayes theorem is effectively rescaling the 200 possible numerators so that the posterior distribution of probabilities add to 1. Thus, the discrete posterior probability distribution for $\phi _w$ consists of the 200 probability values located at the points .0025 to .9975 in steps of .005.

Chechile (2018) showed for $n>24$ that the distribution for $\phi _w$ can be approximated by an adjusted beta distribution. For the large-*n* approach, the posterior distribution is approximated with a beta distribution that has quantiles that are close to the corresponding quantiles of the small-*n* discrete distribution. The resulting shape parameters of the approximating beta distribution are generally not integers.

The function for implementing the Bayesian analysis of the Wilcoxon signed-rank statistic is *dfba_wilcoxon()*. It has two required arguments, which are the *Y*1 and *Y*2 vectors for the two paired variates. In addition to these required arguments, there are six optional arguments. Three of the optional arguments are: *a0*, *b0*, and *prob_interval*, and they have the same meaning and default values as those arguments in the *dfba_binomial()* function. Another optional argument is *method*. This option is only used when the user wants to force one of two approaches for approximating the posterior distribution. One option is *method=“small”* and the other option is *method=“large”*. When this argument is omitted that function uses the discrete small-*n* approach when $n\le 24$ and uses the large-*n* approximation approach when $n>24$. Chechile (2018) explored the *n* value where the small-*n* approach is required, and the *n* value where the large-*n* approach is approximately as accurate as the small-*n* approach. The algorithm for the small-*n* approach runs considerably slower, so the large-*n* approach is desirable for cases where it can be used. Another optional argument for the *dfba_wilcoxon()* function is the *samples* value. This option is only pertinent when the small-*n* approach is used. The number of Monte Carlo samples drawn for each of two hundred candidate values for $\phi _w$ is the value stipulated in the *samples* argument. The default value for *samples* is 30000. The last optional argument is *hide_progress*, which has a preset value of FALSE. When the “small” method is used, the Monte Carlo algorithm can take more than few seconds to complete, so in those cases, the *dfba_wilcoxon()* function displays the percentage complete while running. The argument *hide_progress = TRUE* suppresses progress updates.

As an example of the *dfba_wilcoxon()* consider the following code.

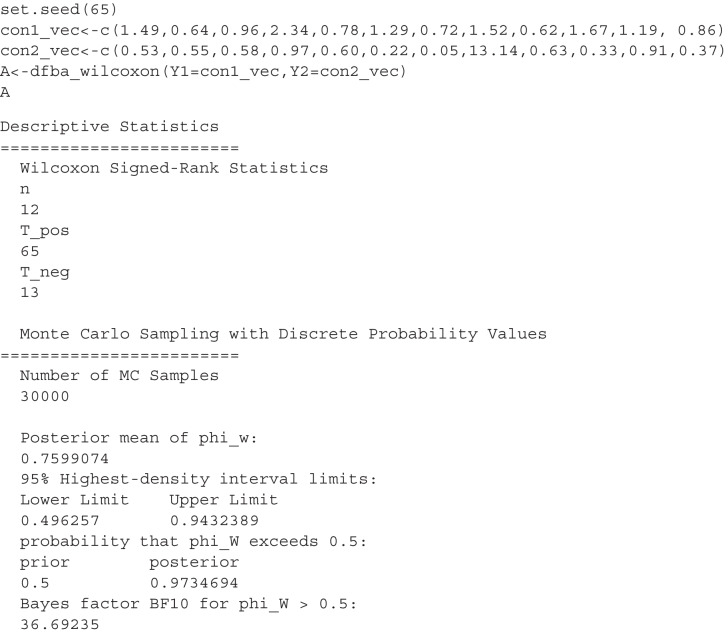



**Fig. 5:**
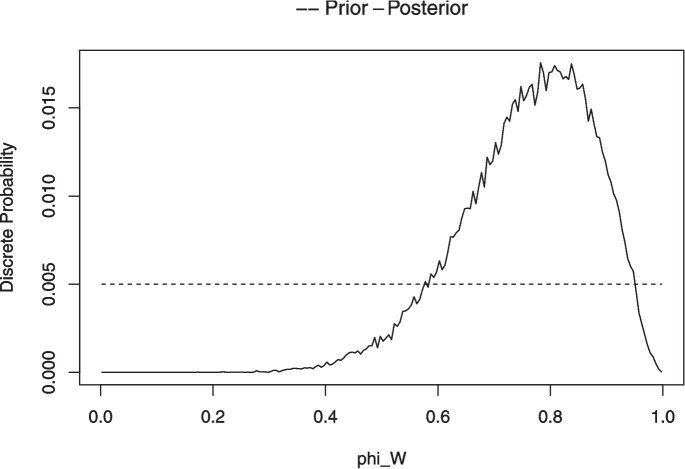
The discrete distributions for the Bayesian Wilcoxon analysis

Because there are only $n=12$ pairs, the *dfba_wilcoxon()* function used the discrete distribution approach (i.e., *method=“small”*). The discrete posterior distribution for $\phi _w$ can be seen by the command *plot(A)* (see Fig. 5).

To compare the discrete algorithm with the large-sample approximation, we can redo the analysis for the above problem by forcing the *dfba_wilcoxon()* function to use *method=“large”*. That is,

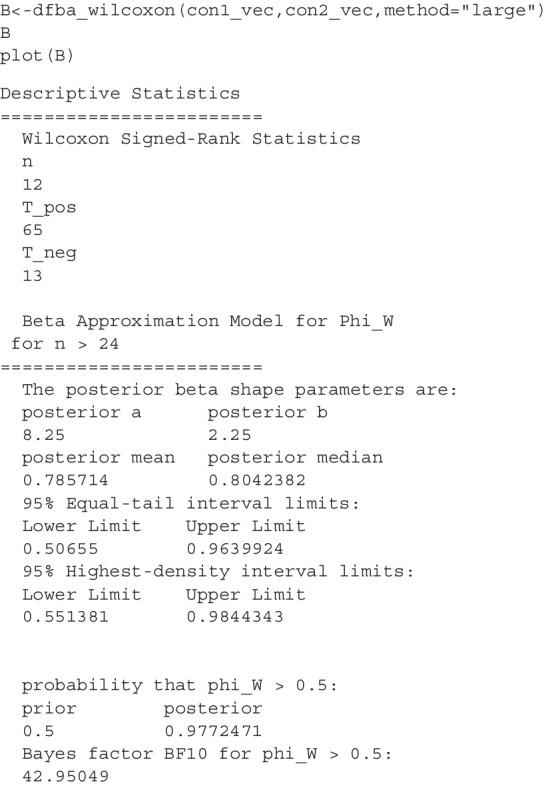



**Fig. 6:**
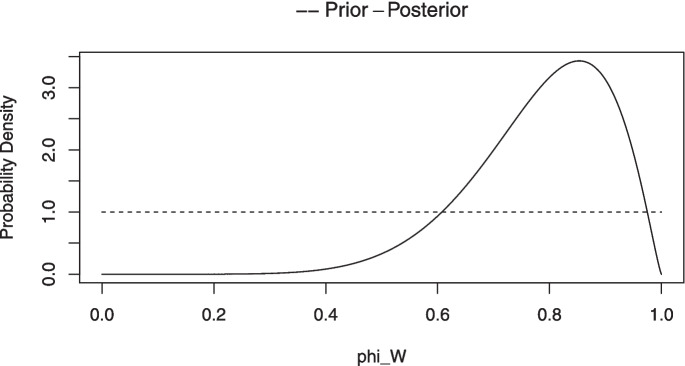
The large-sample beta distribution approximation for the Bayesian Wilcoxon analysis

The instruction *plot(B)* creates the probability-density display for the $\phi _w$ parameter (see Fig. 6).

It is not expected that the second analysis will be accurate because the number of pairs is less than 24, but the analysis nonetheless results in a very similar value for the posterior probability that $\phi _w>.5$ (i.e., .977 for the large-*n* and .973 for the small-*n*).

It is particularly noteworthy to compare the two plots in Figs. 5 and 6. The first plot has a *y* axis of *discrete probability*, and it displays the 200 *discrete probabilities* for $\phi _{wi}=.0025+(i-1)(.005)$ for $i=1,\cdots , 200$; whereas the *y* axis for the second plot is the approximating beta-distribution *probability density* for an infinite number of possible values for the $\phi _w$ parameter on the $[0,\,1]$ interval.

### Bayesian Mann–Whitney analysis

Mann and Whitney (1947) advanced a frequentist procedure for testing if one of two continuous independent random variables was stochastically larger than the other. The *U* statistic is the sample metric for the number of occasions where one variate was larger than the other. To illustrate this idea, let us consider the case where there are $n_C=4$ control-condition observations, which are denoted as $X_{cj}$ for $j=1,\cdots ,4$, and there are $n_E=5$ experimental-condition observations, which are denoted as $X_{ei}$ for $i=1,\cdots ,5$. Suppose the nine measurements were rank-ordered from low to high, and we identify each by either a C or E depending on the condition that the score originated. Let us suppose the ordering was 1C, 2E, 3C, 4C, 5E, 6C, 7E, 8E, and 9E or more simply *CECCECEEE*. There are two *U* statistics – $U_E$ and $U_C$. The $U_E$ metric is the total number of comparisons where an *E* measure is larger than a *C* measure. For the five *E* observations, the first score only beats one control value, but the second *E* score beats three control values, and each of the last three experimental values beats all four of the control observations. Thus, $U_E=1+3+4+4+4=16$. The counterpart $U_C$ statistic for this example is $U_C=0+1+1+2=4$. When there are no ties in rank $U_E+U_C=n_C\,n_E$, but if there are ties, it is possible for sum of the *U* statistics to be less than $n_C\, n_E$. More technically, $U_E$ is generally computed as follows:

10 $$\begin{aligned} U_E= &  \sum _{i=1}^{n_E}\,\sum _{j=1}^{n_C} \delta _E (i,\,j),\end{aligned}$$

11 $$\begin{aligned} \delta _E(i,\,j)= &  \left\{ \begin{array}{ll} 1 & \text{ if } x_{cj}<x_{ei},\\ 0 & \text{ otherwise }, \end{array} \right. \end{aligned}$$

and the $U_C$ statistic is computed as:

12 $$\begin{aligned} U_C= &  \sum _{i=1}^{n_E}\,\sum _{j=1}^{n_C} \delta _C (i,\,j),\end{aligned}$$

13 $$\begin{aligned} \delta _C(i,\,j)= &  \left\{ \begin{array}{ll} 1 & \text{ if } x_{cj}>x_{ei},\\ 0 & \text{ otherwise }. \end{array} \right. \end{aligned}$$

The above equations for $U_E$ and $U_C$ hold regardless of ties.^12^

The ratio $\frac{U_E}{U_E+U_C}$ is the degree to which the *E* values stochastically dominate the *C* condition scores in the sample. The corresponding population parameter $\Omega _E =\lim \frac{U_E}{U_E+U_C}$ is the measure of the degree that the *E* condition is stochastically dominant relative to the *C* condition. There is also an $\Omega _C$ parameter, which must be equal to $1-\Omega _E$. The $\Omega _E$ is a fundamental population characteristic about the relative difference between the *E* and *C* variates. As Chechile (2020a) stated:

$\cdots$ *the population value for* $\Omega _E$ *is a number that must exist. That number contains valuable information about the difference between the two conditions, so if we know the value of* $\Omega _E$*, then it does not matter what the distributional properties are of the* $X_E$ *and* $X_C$ *variates, and it does not matter if the population is infinite or finite. (p. 673).*

Thus, the $\Omega _E$ parameter is a more fundamental comparison between the two independent conditions than say the *t* test, which is only a comparison between the *population means* of the two distributions predicated on the assumption that each variate has a normal distribution with the same variance. Without making those distributional assumptions, the frequentist nonparametric Mann–Whitney test is predicated on the sharp null hypothesis that $\Omega _E=\Omega _C=.5$.

The *dfba_mann_whitney()* function is based on the Bayesian analysis for the Mann–Whitney statistics developed by Chechile (2020a). Instead of evaluating only the point-null hypothesis of $\Omega _E=.5$, the Bayesian analysis assumes a prior distribution for $\Omega _E$. Similar to the analysis for the Wilcoxon signed-rank statistic for small sample sizes, the Bayesian analysis for the Mann–Whitney statistic for small sample sizes approximates the posterior by considering 200 discrete possible values for $\Omega _E$ from .0025 to .9975 in steps of .005. For each candidate value for $\Omega _{Ei}$, for $i=1,\cdots ,200$, there are thousands of random drawings of $n_E$ and $n_C$ scores taken from a pair of distributions that have a population value of $\Omega _{Ei}$.^13^ Given a population value for $\Omega _{Ei}$, the distributional form of the two data-generating distributions does not matter so long as the distributions sampled from have an $\Omega$ value that is equal to $\Omega _{Ei}$. Chechile (2020a) showed how two exponential distributions can always be chosen such that their $\Omega _E$ is equal to $\Omega _{Ei}$. The proportion of the Monte Carlo samples for a given $\Omega _{Ei}$ value that have $U_E$ and $U_C$ statistics that match the observed values for those statistics is taken as an estimate of the likelihood $P(U_E\,|\,\Omega _{Ei})$. Multiplying the likelihood times the prior $P(\Omega _{Ei})$ results in 200 values for the numerator of Bayes theorem. After rescaling the 200 numerators to sum to 1, we arrive at the discrete posterior distribution for $\Omega _E$.

The above discrete-distribution approach becomes increasingly resource-demanding as the sample sizes for the *E* and *C* groups grow. However, the discrete-distribution is unnecessary for larger sample sizes because the posterior distribution can be approximated with a beta distribution with adjusted shape parameters (Chechile, 2020a). This approximation is reasonably accurate when the harmonic mean of the sample size values for the two conditions is greater than or equal to 20.^14^.

The function *dfba_mann_whitney()* implements the Bayesian analysis of the Mann–Whitney *U* statistics. This function has two required argument vectors, which are the *E* and *C* vectors for the two variates. The function also has six optional arguments, which are: *a0*, *b0*, *prob_interval*, *samples*, *method*, and *hide_progress*. These arguments have the same meaning and defaults as for the optional arguments with the same name for the *dfba_wilcoxon()* function(see subsection “Bayesian Wilcoxon analysis”). In the default case, the function decides between the discrete-probability approach and the continuous beta-approximation approach. The decision rule is based on the harmonic mean of the two sample sizes. If the harmonic mean of the sample sizes is greater than 19, then the large-sample approach is used, otherwise the small-sample discrete approach is employed. However, the user can override this decision by stipulating a procedure with the optional *method* argument (i.e., by either setting *method=“small”* or *method=“large”*). The output of the *dfba_mann_whitney()* function provides the means for the *E* and *C* variates, the $n_E$ and $n_C$ values, the $U_E$ and $U_C$ statistics, the posterior mean for $\Omega _E$, the interval estimate for $\Omega _E$, the prior and posterior probabilities for the hypothesis $H_{1}:\,\Omega _{E}>.5$ and the Bayes factor $BF_{10}$ for that hypothesis versus the interval-null hypothesis $H_{0}:\,\Omega _{E}\le .5$.

Consider the following example of the Bayesian Mann–Whitney analysis that employs the small-n approach for the *dfba_mann_whitney()* function:

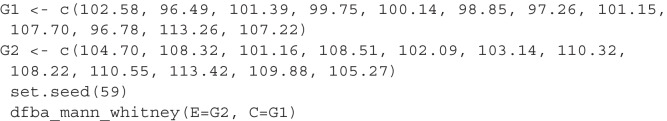



The above code results in the following output:

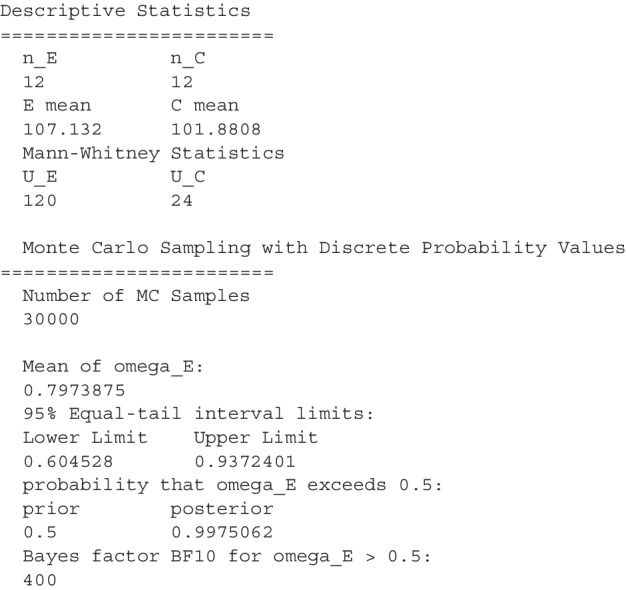



### Bayesian Wilcoxon and Mann–Whitney for $K>2$ variates

Earlier in subsection “The dfba_beta_contrast() function” the binomial analysis was extended to the case where there are a number of separate conditions. In this subsection, similar procedures are described that enable an extension of the Bayesian Wilcoxon and the Bayesian Mann–Whitney analyses for studies where the number of conditions *K* is greater than two.

#### Bayesian Wilcoxon for $K>2$

The standard frequentist nonparametric procedure used when there are $K>2$ within-block or repeated-measures conditions is the Friedman two-way analysis of variance of ranks (Friedman, 1937; Siegel & Castellan, 1988). The Friedman test is another example of a frequentist procedure that assumes the sharp-null hypothesis that there are no differences in the population among any of the *K* conditions. As pointed out in subsection “Theoretical context for the dfba_beta_contrast() function”, this type of null hypothesis has a non-zero *probability density* but has *a probability mass of zero*. From a Bayesian perspective, this null hypothesis is an uninteresting scientific question because we know that the null hypothesis has a probability of zero. Consequently, the Bayesian Wilcoxon signed-rank test when $K>2$ is implemented on a contrast of the conditions. As in subsection “The dfba_beta_contrast() function”, the constraints on the contrast coefficients are: (1) the sum of all the coefficients is zero, (2) the sum of the positive coefficients is $+1$, and (3) the sum of the negative coefficients is $-1$. For a specific contrast, the user can set the *Y1* variate of the *dfba_wilcoxon()* function to be the weighted sum of the condition values that have a positive contrast coefficient and set the $\textit{Y2}$ variate to be the weighted sum of the condition values that have a negative contrast coefficient. Let us illustrate this analysis by means of a specific example. For this example, there are a total of 12 people that are tested in four within-subject conditions. The hypothetical data for this case are shown below.

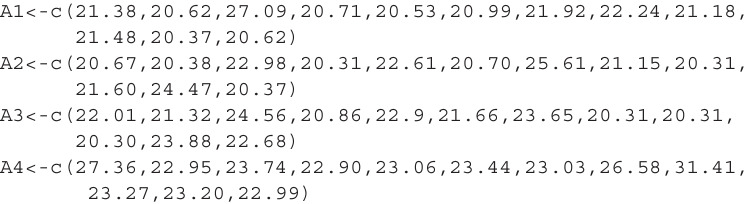



**Fig. 7:**
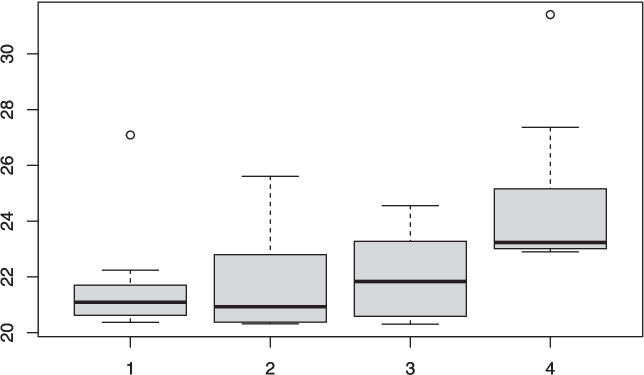
Boxplot of the data for conditions A1, A2, A3, and A4

A boxplot reveals that the data in each condition is positively skewed and that there are two extreme scores (one is condition *A*1 and one in condition *A*4) (See Fig. 7). The boxplot also shows that values in condition *A*4 look to be generally larger than the scores from the other three conditions.

So, the user might be interested in the contrast where the $\psi _i$ coefficients are $(-\frac{1}{3},\,-\frac{1}{3},\,-\frac{1}{3},\,1)$. The following R code will do the Bayesian Wilcoxon analysis for this contrast. 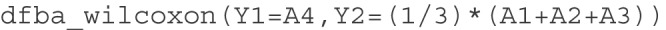


The above analysis results in detecting that the posterior probability for the Wilcoxon $\phi _w$ parameter being greater than .5 is approximately .994. Also, the Bayes factor for the *A*4 condition being greater than the average of the other three conditions is greater than 150.

For the above example, the user might also consider two other orthogonal contrasts where the coefficients are: $(-1,\,\frac{1}{2},\,\frac{1}{2},\,0)$ and $(0,\,-1,\,1,\,0)$. Neither of these two analyses result in a large Bayes factor value; these Bayes factors are respectively 2.0 and 3.4. So, the data are insufficient for making a decision about either the null or the alternative hypothesis for these two contrasts by the rule-of-thumb of limiting scientific claims to cases where the Bayes factor equals or exceeds 19 (see Appendix A).

#### Bayesian Mann–Whitney for $K>2$

The Kruskal–Wallis test is the standard frequentist nonparametric procedure when there are $K>2$ independent groups (Kruskal & Wallis, 1952; Siegel & Castellan, 1988). This test (like the Friedman procedure discussed in the previous subsection) is another frequentist statistical procedure that examines the sharp-null hypothesis that each of the conditions in the population are identical. As argued above, this sharp-null hypothesis has a probability mass of zero. Consequently, in the Bayesian analysis there is instead a variation on the Bayesian Mann–Whitney analysis that examines a contrast by the pooling of groups. To illustrate the application of the Bayesian Mann–Whitney analysis for an experiment where there are more than two conditions, let us reexamine the above example for the *A*1, *A*2, *A*3, and *A*4 score values under the revised assumption that there are 12 *separate* participants in each condition. Since the variates in the four conditions are no longer paired in this revised example, a contrast comparison is done differently from the above Wilcoxon analysis. For the Mann–Whitney procedure, the conditions are grouped for the *E* and *C* variates. So, for the comparison between *A*4 versus the other three conditions, the *E* variate is set equal to *A*4 while the *C* variate is set equal to the combined data in the first three conditions. The sample sizes for the resulting *E* and *C* variates are not equal, but these variates need not be equal for the Mann–Whitney procedure. Consequently, the counterpart to the three contrasts examined in subsection “[Sec Sec27]” are obtained with the following code.

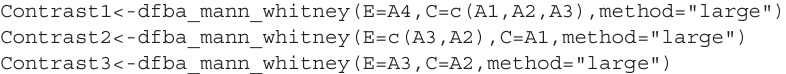



The analyses from the above R function calls resulted in finding a high Bayes factor greater than 30, 000 for the first contrast comparing the *A*4 condition with the other three conditions, and also resulted in finding very low Bayes factor values for the other two contrasts (i.e., $BF10 \approx 1.7$ for the second contrast and $BF10 \approx 2.1$ for the third contrast).^15^

## Bayesian and *t* power simulations

### Preliminary remarks about power

The concept of power originated from the frequentist framework for null-hypothesis testing. Setting the $\alpha$ level creates a *rejection region* for the test statistic. The test statistic has a probability *over repeated samples* of $\alpha$ for landing in the rejection region when the *sampling is based on the null hypothesis*. However, if the sampling is done instead from a specific value for the parameter (i.e., a specific value for the parameter in the range of possible values for the alternative hypothesis), then the probability for the test statistic landing in the rejection region over repeated samples is the *conditional power*. Power equals $\alpha$ when the population parameter is equal to the null value (i.e., when the null and specific alternative are the same), and power increases as a function of the separation between the null value and the alternative value. Moreover, as the sample size increases, the power increases for a fixed separation between the null and alternative values. Hence, frequentist power is useful as a research design tool for planning a forthcoming experiment that will employ a frequentist statistical test.

Unfortunately, frequentist power is a widely misunderstood and misused concept in actual statistical practice. Hoenig and Heisey (2001) criticized the widespread use of *observed power or post hoc power* by frequentist data analysts. Observed power uses the test statistic found from an actual experiment to estimate the population parameter for the alternative hypothesis and then computes a conditional power value. Hoenig and Heisey (2001) showed that the observed power is a function of the *p* value. Observed power is not a frequentist probability because *it does not have a relative frequency*. The use of observed power is thus a variation on the *p* value fallacy that was discussed in Section “Introduction”. There is no frequentist power after an experiment has been conducted; rather *power is a feature of the research sampling plan*.

In a Bayesian analysis of actual data, observed power or post hoc power in the frequentist sense is not needed, and it is not used. Yet the design of forthcoming research does require experimental-design decisions regardless of the statistical framework that the researcher plans to use for the data analysis. If a Bayesian analyst studied a research plan and obtained many different random samples of data via Monte Carlo simulation, then a Bayesian counterpart to frequentist power could be found. Bayesian power in this paper is estimated by the proportion of the separate Monte Carlo-generated data sets that would find a robust effect for a Bayesian distribution-free analysis.^16^ Thus, Bayesian power can inform researchers about the design for a forthcoming study that will employ a distribution-free Bayesian analysis.

It is a common frequentist practice to ascertain the sample size that would result in some desired power on a parametric *t* test for a specific effect size. However, if an investigator were instead planning to do a Bayesian distribution-free analysis, then the same type of sample size calculation is pertinent. There are two functions in the DFBA package that estimate Bayesian (distribution-free) power for various sample sizes where the data originate from any one of nine different probability models. The DFBA power functions compute the proportion of the Monte Carlo samples where the appropriate distribution-free analysis (i.e., either the Bayesian Wilcoxon or the Bayesian Mann–Whitney) detects with a high probability a difference for some specific effect size. The DFBA functions also compute the corresponding frequentist power based on a parametric *t* test. While the frequentist *t* power values are included as a comparison reference, these *t* power values are quite general and can be used for some probability models that are beyond those provided by the existing frequentist R power functions (e.g., the Champely (2020) R *pwr* package, which is based on the Cohen (1988) power analysis). Thus, these DFBA power functions are designed to be useful to researchers who may need to justify to a wide audience the rationale for the sample-size decision for a proposed experiment (such as for a grant proposal or for a pre-registered paper).

### DFBA power functions

To guide researchers in planning studies that have the desired power level, the DFBA package has three useful functions: *dfba_sim_data()*, *dfba_bayes_vs_t_power()*, and *dfba_power_curve()*. The *dfba_sim_data()* function generates data for two variates from nine different probability models, and based on the generated data, the function computes the Bayesian posterior probability from a distribution-free analysis for the alternative hypothesis, and for a reference, it computes the frequentist *p* value from a *t* test. The nine probability models are: (1) normal, (2) Weibull, (3) Cauchy, (4) lognormal, (5) chi-squared, (6) logistic, (7) exponential, (8) Gumbel, and (9) Pareto.^17^ If the forthcoming experiment is for paired variates, then the *dfba_sim_data()* function computes the frequentist *p* value for a paired *t* test, and it also computes the posterior probability that the Bayesian Wilcoxon $\phi _w$ parameter is greater than .5. If the forthcoming experiment is for two independent groups, then the *dfba_sim_data()* function computes the *p* value from a two-sample *t* test, and the function also computes the posterior probability that the Mann–Whitney $\Omega _E$ parameter is greater than .5. Importantly, the *dfba_sim_data()* function *does not estimate power*. Instead, it is a helper function that is used repeatedly by either the *dfba_bayes_vs_t_power()* function or the *dfba_power_curve()* function. Power values are estimated from the Monte Carlo sampling done by those two other functions. Since it is unlikely that experimental scientists will need to directly use the *dfba_sim_data()* function, the details about this function are omitted here.^18^ All the details about power estimation are described in subsections “The dfba_bayes_vs_t_power() function” and “The dfba_power_curve() function”.

For any given probability model and research design, the conditional power depends on the separation between the two distributions in the population, and the sample size. Each DFBA power function varies one of those two factors for a fixed value of the other factor. For the *dfba_bayes_vs_t_power()* function, the separation between the two conditions must be set to a fixed value so that the power can be estimated for 11 different sample sizes. These 11 sample sizes vary from a low of $n_{min}$ to a high of $n_{min}+50$ in steps of 5. For the *dfba_power_curve()* function, the sample size *n* is fixed by the user, and the power is estimated for 21 separation values, which start at a minimum of 0 and go to a maximum of $20\times \Delta _{step}$; the value for $\Delta _{step}$ is a function argument called *delta_step*. These two power functions are described and illustrated in the next two subsections.

#### The dfba_bayes_vs_t_power() function

The *dfba_bayes_vs_t_power()* function has three required arguments and nine optional arguments. The three required arguments are: *delta*, *model*, and *design*. The *delta* argument is the separation between the two population distributions. The *model* argument is a user-specified probability model for the variates, and it must be a (case sensitive) text string from the following list of options: *“normal”, “weibull”, “cauchy”, “lognormal”, “chisquare”,“logistic”, “exponential”, “gumbel”, “pareto”*. The *design* argument is the statistical design of the forthcoming study for the two variates, and this argument must be either the string *“paired”* or the string *“independent”*. The nine optional arguments along with their default values are the following: *n_min=20*, *a0=1*, *b0=1*, *effect_crit=.95*, *shape1=1*, *shape2=1* *samples=1000*, *block_max=0*, and *hide_progress=FALSE*. The *n_min* argument is the initial sample size for each of the two variates. The *dfba_bayes_vs_t_power()* function computes power for 11 samples sizes that begin with the value of the *n_min* argument, and it increases in steps of 5.^19^ The *a0* and *b0* arguments are the shape parameters for the beta distribution that is used for the prior for either the $\phi _w$ parameter (if *design=“paired”*) or the $\Omega _E$ parameter (if *design=“independent”*). The *effect_crit* argument is the threshold probability for the alternative hypothesis in the Bayesian analyses to be counted as a detection for the distribution-free metric; also one minus *effect_crit* is the frequentist $\alpha$ value for a significant frequentist *t*-test. The *shape1* and *shape2* arguments are the model shape parameter values for respectively the first and second condition variates.^20^ The *samples* argument is the number of Monte Carlo data sets generated for each sample size. The *block_max* optional argument is used to add a block effect in addition to the effect of the separate conditions. This argument has a default setting of no block variation (e.g., no systematic differences between people). A major advantage for a paired design is that the effect of individual differences is removed because for each person (or block) the measurements are taken in both of the two conditions. However, the effect of block variation cannot be removed for the independent-groups design. Later in this subsection an example using the *block_max* argument is provided to see the effect of block variation on the power for the independent-group design. Finally, the optional argument of *hide_progress* provides the user with a way to suppress the progress report produced by the *dfba_bayes_vs_t_power()* function as it does the Monte Carlo simulations for the power estimates. To suppress the progress report the user needs to set *hide_progress* argument to *TRUE*.

As an example of the *dfba_bayes_vs_t_power()* function, suppose the user is interested in determining a sample size that would result in approximately 90% of the samples being decided in favor of the alternative interval hypothesis when the separation between the conditions is $\Delta =.45$ and where the variates are sampled from normal distributions with a standard deviation of 1. Furthermore, suppose the user wants to see the conditional power values for sample sizes in the 50 to 100 range. The following function call provides power estimates over that range of sample sizes.

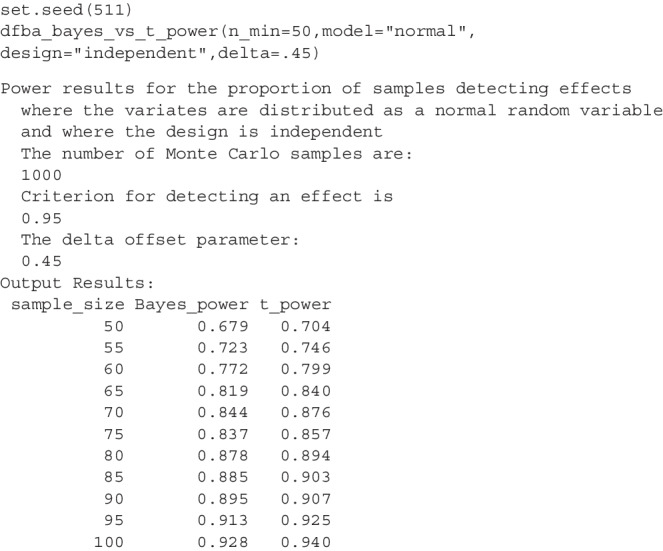



Additional runs of the function with a value of 10000 for *samples* and with other values for *n_min* resulted in finding a value sample size of $n_b =91$ for the Bayesian power being about .9. Those additional runs also resulted in finding a value $n_t=86$ for the frequentist *t* power being about .9. The ratio $n_t/n_b$ is an estimate of the *relative power efficiency* of the Bayesian distribution-free Mann–Whitney procedure compared to the frequentist *t* test. A relative efficiency less than 1.0 favors the parametric *t* test procedure, and a power efficiency greater than 1.0 favors the Bayesian distribution-free procedure. For the case of sampling from a normal distribution for two independent groups, the relative power efficiency is estimated as 86/91 or about .95. Hence, for the case where the assumptions that underlie the *t* of normally distributed variates are valid, the cost for doing a Bayesian distribution-free approach is relatively small (i.e., five extra observations per condition when the study is designed to have power of about .9).

The *dfba_bayes_vs_t_power()* function can also be used to examine the power efficiency for other distributions. For example, in Table 3 the values for $n_b$, $n_t$, and the power efficiency are provided for a range of distributions where $\Delta =.45$ and where the design goal is to have power be .9. Note for all the distributions other than the normal, the estimated power efficiency is greater than 1.0; thus there is a benefit to using the distribution-free Bayesian analysis. If the user’s data are not sampled from normal distributions, then the parametric *t* test is likely to have less power since the *t* test depends crucially on the validity of the parametric assumptions. Note even for the logistic distribution when the scale factor is .551, which corresponds to a distribution that is an excellent approximation to a normal distribution, the power efficiency is still greater than 1.0.^21^

**Table 3:** Sample sizes $n_b$ and $n_t$ for respectively the Bayesian power and *t* power to be .9 as a function of model, $\alpha =.05$, $\Delta =.45$ and where the design is paired

model	$n_b$	$n_t$	rel. efficiency $n_t/n_b$
Normal	91	86	.95
Lognormal	101	143	1.42
Weibull (shape$=.8$)	93	176	1.89
Gumbel	130	140	1.08
Exponential	65	88	1.35
$\chi ^{2}$ ($df=2$)	234	340	1.45
Logistic (scale$=.551$)	85	86	1.01
Pareto (shape$=1.301$)	56	1411	25.2
Cauchy (scale$=1$)	578	NA	NA

There were 10000 Monte Carlo samples for each case

It is also notable in Table 3 that we were unable to find a sample size for the Cauchy distribution where the frequentist t-based power was .9. The Cauchy distribution is known as a pathological probability model where the moments of the distribution are not defined (Johnson et al., 1994). The classical *t* test is particularly ill-suited for this distribution. Classical *t* power for the Cauchy is insensitive to sample size. For example, for $\Delta =.45$ the t-power for *n* in the range of 20 to 70 is approximately .065, but the corresponding t-power for the range of 520 to 570 is still only .068. To get a t-power value of .9 for the Cauchy distribution, the $\Delta$ value for the offset between the two distributions needs to be 20 rather than .45. Hence, for the cases in Table 3 where $\Delta =.45$ there was no sample size where t-power was .9. Yet, in contrast to t-power, the Bayesian power is relatively well behaved for the Cauchy model.

Block variation can be added to the power analyses with the inclusion of a non-zero value for the *block_max* argument. With a non-zero value for *block_max* argument there is a separate random variability component added to the response metric due to block differences. This feature is important for the independent-groups design.

**Table 4:** Sample sizes $n_b$ and $n_t$ for the respective Bayesian power and *t* power being .9 with $\alpha =.05$ for $\Delta =.45$, as function of model, design, and the *block_max* factor as measured by the ratio of the variance of blocks to treatment variance

model	$\frac{V(blocks)}{V(\Delta )}$	$n_b$	$n_t$	$\frac{n_t}{n_b}$
normal	0	91	86	.95
	1/9	93	87	.94
	1	96	89	.93
	4	111	103	.93
	9	136	126	.93
	36	271	242	.89
Weibull(.8)	0	50	175	3.50
	1/9	50	175	3.50
	1	60	179	2.98
	4	91	195	2.14
	9	131	213	1.63
	36	294	325	1.11

Each power is based on 10000 Monte Carlo samples

In Table 4 there are six levels of block variability examined (i.e., zero and five cases where *block_max* is non-zero). The block effect is a random score from a uniform distribution that has *block_max* as the range of the distribution. For a uniform distribution, the variance is equal to 1/12 times the square of the range. So, if *block_max*$=\sqrt{3}\Delta$, then the variance due to blocks is $V(blocks)=\frac{3\Delta ^{2}}{12}=\frac{\Delta ^{2}}{4}$. The treatment variance (i.e., due to the offset of $\Delta$ between the two conditions) is $V(\Delta )=\frac{\Delta ^{2}}{4}$. So, if the *block_max*$=\sqrt{3}\Delta$, the ratio $\frac{V(blocks)}{V(\Delta )}$ would be 1. The values for *block_max* listed in Table 4 are expressed in terms of the ratio $\frac{V(blocks)}{V(\Delta )}$. The independent-groups research design is used for each case. The table examines the standard normal distribution and the Weibull distribution that has a shape parameter of .8. For all the cases for the Weibull distribution, regardless of the level of the blocking, there is an advantage of greater power for the Bayesian distribution-free analysis to that of the classical *t* test. Thus, the cost of the reduced power is small when the data are distributed as a normal, but when the data are not normally distributed, the classical *t* test has generally less power than the Bayesian distribution-free analysis.

#### The dfba_power_curve() function

The *dfba_power_curve()* function computes conditional-power values for various separations between the two population variates for a fixed sample size *n*. The *dfba_power_curve()* function has two required arguments and ten optional arguments. The two required arguments are *model* and *design*, which have the same meaning as the arguments in the *dfba_bayes_vs_t_power()* function with those names. The ten optional arguments along with their default values are: *n=20*, *a0=1*, *b0=1*, *delta_step=.05*, *effect_crit=.95*, *shape1=1*, *shape2=1* *samples=1000*, *block_max=0*, and *hide_progress=FALSE*. The *n* argument is the sample size for each power calculation^22^ The *delta_step* argument is the increment for the separation between the two variates for consecutive cases. The *dfba_power_curve()* function computes 21 power values for separations between the two conditions that vary from zero to 20 times *delta_step*. All the other optional arguments have the same meaning and default values as the arguments in the *dfba_bayes_vs_t_power()* function that have the same name. The following instructions illustrate this function.

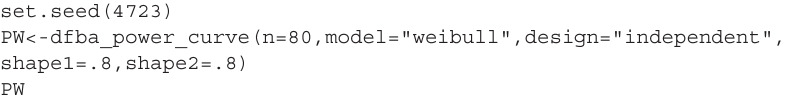

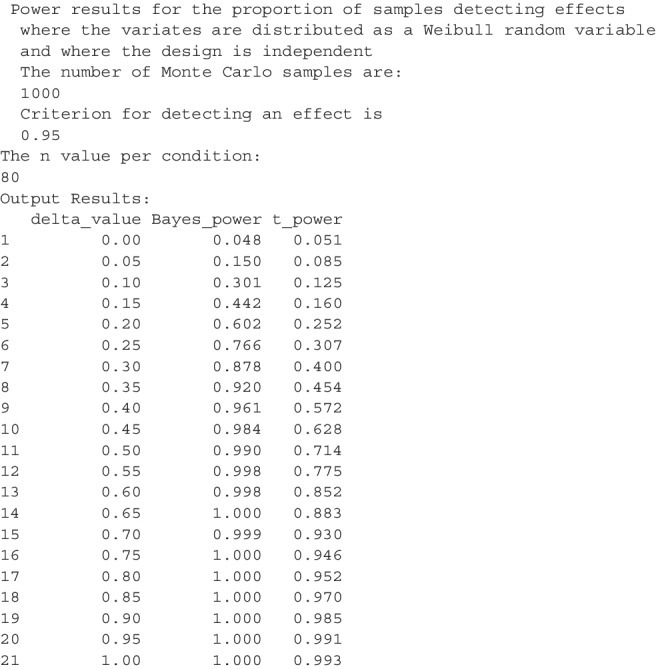





**Fig. 8:**
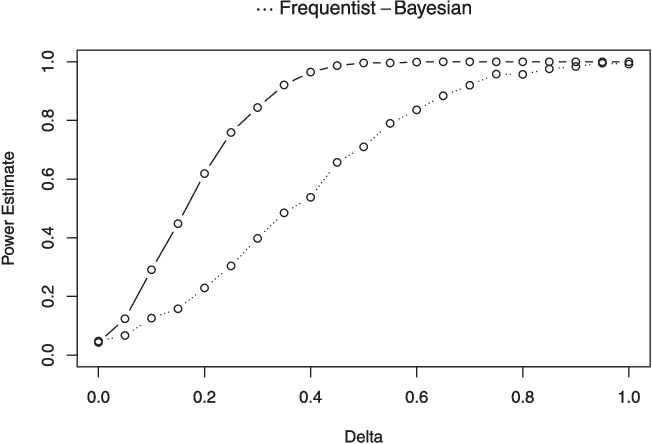
The power curve for $n=80$ for data that are distributed from two independent Weibull distributions each with a shape parameter of .8. The figure is produced by the DFBA package plot method for the *dfba_power_curve()* function

The above call for the *dfba_power_curve()* function created a new R object called *PW*. Thus, the instruction *plot(PW)* produced the display shown in Fig. 8.

It is also possible that the user might prefer to plot the power data in a different fashion that employs some other plotting functions and tools available in R. As an example, suppose the following instructions are added to extract the plotting data from the newly created R object. 
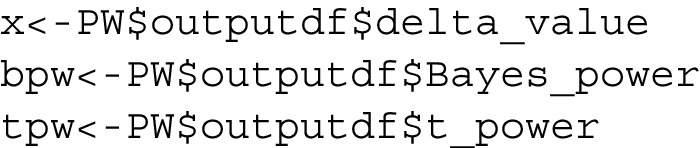


**Fig. 9:**
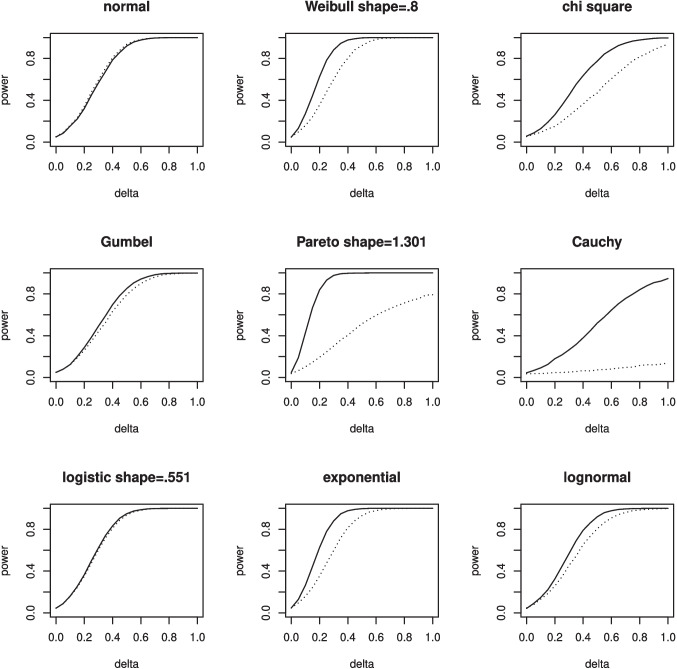
Power curves for $n=80$ for nine probability models on a common scale. Bayes power is the *solid line*, and *t* power is the *dotted line*. The data for each plot is obtained from the *dfba_power_curve()* function with *delta_step=.05*, *design=“independent”*, *effect_crit=.95*, *block_max*$=0$, and *samples=10000*

The above code creates three new R vectors called **x**, **bpw**, and **tpw** from an internal R dataframe called *outputdf* that was produced with the call of the *dfba_power_curve()* function. These vectors are, respectively, the separation between the distributions, the Bayesian-power values, and the *t*-power values. These vectors can be useful if the user wants to use other R functions to create displays that combine the results from separate power analyses. For example, Fig. 9 is a display of power plots for nine probability models where $n=80$. Each of the panels in the figure came from separate R objects generated by a specific call of the *dfba_power_curve()* function.

Figure 9 makes clear that the Bayesian distribution-free analysis has trivially less power than that of the *t* test when the normative Gaussian model is the basis of the data generated, but for the other distributions shown in the figure, the Bayesian distribution-free power exceeds the *t* power, although that difference is slight for the logistic model when the shape parameter is .551. Overall, the figure illustrates the strength of the Bayesian distribution-free methods, and it also illustrates some cases where the frequentist *t* test is clearly non-optimal.

## Distribution-free bivariate statistical association

The focus in this section is on Bayesian distribution-free measures of statistical association between bivariate measures. The statistical properties of standard product-moment correlation are based on the parametric model of homogeneous Gaussian measurement error, which is unlikely to be precisely true. Moreover, the correlation coefficient is sensitive to outlier scores for either variate, so it is not a robust statistical measure of association.

There are two DFBA functions that deal with bivariate association. One is the *dfba_bivariate_concordance()* function, and the other is the *dfba_gamma()* function. These functions are described separately in the next two subsections.

### Concordance proportion $\phi _c$ and the Kendall $\tau _A$ coefficient

#### Background for the $\phi _c$ and $\tau _A$ parameters

Several rank-based correlation measures are alternatives to the product–moment correlation. All the rank-based correlation metrics convert the *Y* scores to rank values $R_Y$ and they convert the *X* scores to rank values $R_X$. Also these correlation coefficients are similar to the Pearson correlation in that they are a measure of the association between two variates on a $[-1,\,1]$ scale. Typically three rank-based correlations have been used: the Spearman $r_S$, the Kendall $\tau _A$, and the Kendall $\tau _B$.^23^ Our attention in this subsection is focused on the Kendall $\tau _A$ because it has a straightforward Bayesian analysis. Nonetheless, a few comments are in order here about the other two correlations.

First, the Spearman $r_S$ measure is simply the product-moment correlation between the $R_Y$ and $R_X$ scores. Although this metric is robust to the influence of outliers, it still requires some parametric assumptions about the errors in the rank values (Caruso & Cliff, 1997). If the reader wishes to perform a Bayesian analysis of the Spearman correlation, then the user can rank order the data and use a Bayesian parametric correlation analysis (e.g., the R *BayesFactor* package).

Second, only one of the two Kendall correlations is used in the DFBA function because the $\tau _B$ correlation is flawed when there are ties. Unfortunately, the $\tau _B$ measure is the more widely used metric. For example, the *method = “kendall”* argument in the *cor()* and *cor.test()* functions in the R *stats* package computes $\tau _B$ rather than $\tau _A$. If there are no tied values in the data, then $\tau _B=\tau _A$, and there is no problem.

So, what is $\tau _A$, and how is it computed? The definition of this metric is more clear in the context of several simple examples based on the data shown in Table 5. For the first example, the two variates are *X* and *Y*, whereas for the second example, the variates are $X_2$ and $Y_2$. For example 1, the columns labeled *RX* and *RY* are the rank values for the respective *X* and *Y* variates. Between any two points, the two variates have a variation that is either *concordant* or *discordant*. The change between two points is concordant if the change in *Y* and the change in *X* are both in the same direction; the change is discordant when the direction of the *Y* change is the opposite direction from the change in *X*. The formal criterion for a concordant change for points *i* and *j*, where $i\ne j$, is $sgn(RY_i - RY_j)=sgn(RX_i -RY_j)$; whereas the definition of a discordant change is $sgn(RY_i - RY_j)=-sgn(RX_i -RY_j)$. For example, between points $P_2$ and $P_1$ the sign of the change in *RY* is positive (i.e., $3-2=+1$) and the corresponding change in *RX* is also positive (i.e., $2-1=+1$). Since the signs for the changes in the ranks are the same for these two points, this comparison is a concordant relationship between the variates. A similar comparison between points $P_3$ and $P_1$ results in a discordant pattern between the variates (i.e., a negative change for *RY* but a positive change for *RX*). When there are no ties for either variate, there are $\frac{n(n-1)}{2}$ comparisons. Thus, for the first example, there are $\frac{5 \cdot 4}{2}=10$ comparisons where there are $n_c=7$ concordant changes and $n_d=3$ discordant changes. In general, the definition of $\tau _A$ is

14 $$\begin{aligned} \tau _A = \frac{n_c-n_d}{n_c+n_d}. \end{aligned}$$

**Table 5:** Two examples for illustrating the Kendall $\tau _A$ correlation

Point #	*X*	*Y*	*RX*	*RY*	$X_2$	$Y_2$	*RX*2	*RY*2
1	-5.6	1.5	1	2	-5.6	1.5	1	2
2	-.5	4.7	2	3	1.3	4.7	2.5	3
3	2.1	1.1	3	1	1.3	1.1	2.5	1
4	3.6	6.2	4	5	3.9	6.1	4.5	4.5
5	4.1	5.9	5	4	3.9	6.1	4.5	4.5

If $n_d=0$, $\tau _A=1$, but if $n_c=0$, then $\tau _A=-1$. Also if the two variates have a correlation of zero, then $n_c=n_d$. For example 1, the Kendall $\tau _A=\frac{7-3}{7+3}=0.4$.

The second example from Table 5 is designed to illustrate the complicating factor of tied values. Note for *RX*2 there are two clusters of tied values (i.e., points $P_2$ and $P_3$ are tied as are points $P_4$ and $P_5$). If there is a cluster of $t_X$ tied values, then there are $\frac{t_X (t_X-1)}{2}$ lost comparisons. In general, the total number of lost comparisons due to ties for the generic *X* variate is denoted as $T_X$, which is given as

$$\begin{aligned} T_X =\sum _i^{m_X} \frac{t_{X_i} (t_{X_i}-1)}{2}, \end{aligned}$$

where $m_X$ is the number of tied clusters for *X*, and $t_{X_i}$ is the number of scores in the *i*th cluster of tied values. Thus, for the second example, $m_X=2$ with $t_{X_1}=t_{X_2}=2$; hence, $T_X=\frac{2\cdot 1}{2}+\frac{2\cdot 1}{2}=2$. In a similar fashion, the number of lost comparisons due to ties for the *Y* variate is $T_Y$ where

$$\begin{aligned} T_Y =\sum _i^{m_Y} \frac{t_{Y_i} (t_{Y_i}-1)}{2}, \end{aligned}$$

where $m_Y$ is the number of clusters that are tied on the *Y* variate and $t_{Yi}$ is the number of scores tied for the *i*th cluster. For the second example, $m_Y=1$ and $t_{Y_1}=2$, so $T_Y=1$. Finally, we need to consider cases where there are clusters of points that are tied on both the *X* and *Y* variates. Please note that for the second example, the points $P_4$ and $P_5$ are tied on for both variates. The total number of comparisons that are multiply tied is denoted as $T_{XY}$ and is computed as

$$\begin{aligned} T_{XY} =\sum _i^{m_{XY}} \frac{t_{XY_i} (t_{XY_i}-1)}{2}, \end{aligned}$$

where $m_{XY}$ is the number of clusters that are tied values for both variates. Thus, for the second example, $m_{XY}=1$, and $t_{XY}=2$, so $T_{XY}=1$. The total number of comparisons that can be compared is $n_c+n_d$, and this quantity is equal to

15 $$\begin{aligned} n_c+n_d = \frac{n (n-1)}{2} - T_X - T_Y +T_{XY}. \end{aligned}$$

The $T_{XY}$ term is added in Eq. 15 to correct for an over-correction of lost comparisons in the subtraction of $T_X$ and $T_Y$. Note further that it follows from Eqs. 14 and 15 that

16 $$\begin{aligned} \tau _A =\frac{n_c-n_d}{\frac{n (n-1)}{2} - T_X - T_Y +T_{XY}}. \end{aligned}$$

Thus, the number of comparisons for the second example from Eq. 15 is $\frac{5 \cdot 4}{2}-2-1+1=8$. Since this example is relatively simple because of the small number of bivariate pairs, we can also count the number of comparisons that can be categorized as either concordant or discordant and arrive at the same answer. That is, the possible comparisons for example 2 are between the following pairs of points: $(P_1,\,P_2)$, $(P_1,\,P_3)$, $(P_1,\,P_4)$, $(P_1,\,P_5)$, $(P_2,\,P_4)$, $(P_2,\,P_5)$,$(P_3,\,P_4)$, and $(P_3,\,P_5)$. Of these eight comparisons all are concordant except for the $(P_1,\,P_3)$ comparison. Thus, for this example, $\tau _A=\frac{7-1}{7+1}=0.75$.

The above two examples also provide a way to see the difference between the $\tau _A$ and $\tau _B$ correlations. Kendall (1945, 1955) defined the correlation coefficient that has come to be called $\tau _B$ as

17 $$\begin{aligned} \tau _B = \frac{n_c-n_d}{\sqrt{(\frac{n(n-1)}{2}-T_X)}\sqrt{(\frac{n(n-1)}{2}-T_Y)}}. \end{aligned}$$

So, for the second example, $\tau _B$ is $\frac{7-1}{\sqrt{(10-2)(10-1)}}=\frac{\sqrt{2}}{2}=0.7071$, which is not the correct value of .75 as computed above. Unfortunately, $\tau _B$ is nonetheless the most commonly used coefficient associated with Kendall. Kendall (1945) did not provide a proof for his correction formula. His only justification was to propose a correction for ties in a fashion to be similar to Daniels (1944), who was trying to treat all the correlation coefficients in terms of a permutation framework. Moreover, using his own example for the correction for ties on page 35 in the Kendall (1955) book, the sample correlation results in an error. In his example, there are ten bivariate pairs where the sample correlation must be 1 because *all possible comparisons between the points are concordant in his example!* Yet he reported after using Eq. 17 that $\tau _B=\frac{33}{\sqrt{(\frac{45}{2}-9)}\sqrt{(\frac{45}{2}-4)}}=\frac{33}{\sqrt{(36\cdot 41)}}=0.85896$, which is obviously incorrect. However, if Eq. 16 were used instead, then the sample correlation would correctly be $\frac{33}{45-4-9+1}=\frac{33}{33}=1$.^24^ Thus, one should be warned that the use of the base R correlation for the Kendall tau (i.e., the function call *cor(x,y,method=“kendall”)*) is based on the flawed Eq. 17 rather than the $\tau _A$ equation. The DFBA package provides a function that correctly computes the frequentist $\tau _A$ statistic along with a Bayesian analysis of the bivariate association, and it does *not* include the $\tau _B$ statistic in any of its functions (for all of the reasons given in this section).

A Bayesian analysis for the $\tau _A$ coefficient was developed by Chechile (2020b); a more streamlined discussion of this approach along with an extensive application from the behavioral sciences can be found in Chechile and Barch (2022). A key concept in this analysis is the linkage of the $\tau _A$ parameter with the population parameter of the proportion of concordant bivariate changes among the points for the two variates. The concordance proportion $\phi _c$ is defined as the population limit of $\frac{n_c}{n_c+n_d}$. The population value for $\tau _A=2\phi _c-1$. In the Bayesian analysis, a prior and posterior distribution is generated for $\phi _c$, and thus there is a corresponding posterior distribution $\tau _A$ because by definition $2\phi _c-1=\frac{2n_c}{n_c+n_d}-\frac{n_c+n_d}{n_c+n_d}=\frac{n_c-n_d}{n_c+n_d}=\tau _A$.

In the Bayesian analysis the observed frequencies for $n_c$ and $n_d$ for the comparisons among the points are considered fixed, and the value for $\phi _c$ is considered a random variable with a prior distribution.^25^ Because each pair of points where the two variates can be compared results in a binary outcome (i.e., it is either concordant or discordant), the data are generated by a Bernoulli process.^26^ Thus, the Bayesian likelihood after the data are sampled is

18 $$\begin{aligned} \ell (n_c, n_d\,|\,\phi _c) \propto \phi _{c}^{n_c}(1-\phi _c)^{n_d}. \end{aligned}$$

As for all Bernoulli processes, a prior for $\phi _c$ that is a beta density function with shape parameters $a_0$ and $b_0$, results in a posterior distribution that is also a beta density function with the shape parameters being $a_0+n_c$ and $b_0+n_d$.

#### The *dfba_bivariate_concordance()* function

The Bayesian analysis for $\phi _c$ is implemented via the *dfba_bivariate_concordance()* function, which has two required arguments and four optional arguments. The two required arguments are *x* and *y*, which are the respective vectors of values for the *X* and *Y* measures. The user needs to be sure that scores *x*[*i*] and *y*[*i*] scores are the two paired values for the *i*th block. Three of the four optional arguments are *a0*, *b0*, and *prob_interval*, and these arguments have the same meaning as the arguments with the same names in the *dfba_binomial()* function that was discussed in subsection “The dfba_binomial() function”. The fourth optional argument is *fitting.parameters*, and it not applicable for most correlational problems. This fourth optional argument is used for a goodness-of-fit test of a mathematical model of a univariate measure; this type of application is discussed in subsection “Distribution-free goodness-of-fit of a scientific model”.

As an example of using the *dfba_bivariate_concordance()* function, see the following code and printed output:

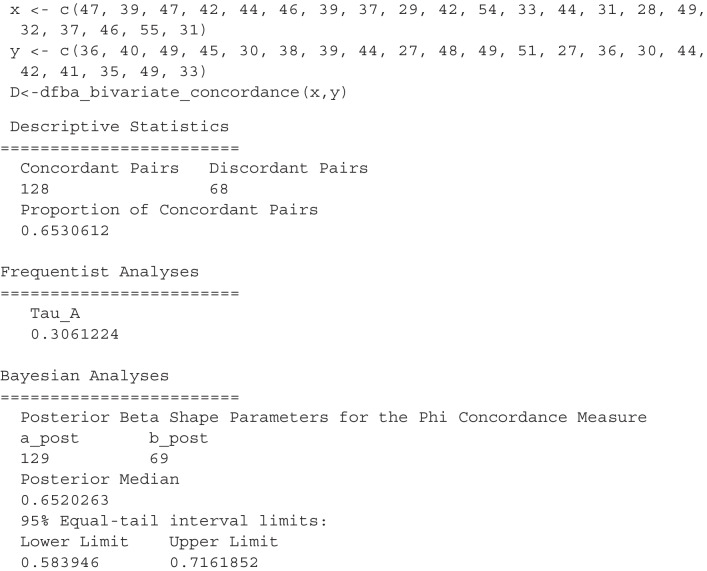



A plot of the prior and posterior distributions for $\phi _c$ can be obtained from the instruction *plot(D)*. Since the posterior distribution for $\phi _c$ is a beta variate, the Bayes factor $BF_{10}$ for the hypothesis that $\phi _c>.5$, can be found by using the following function call:

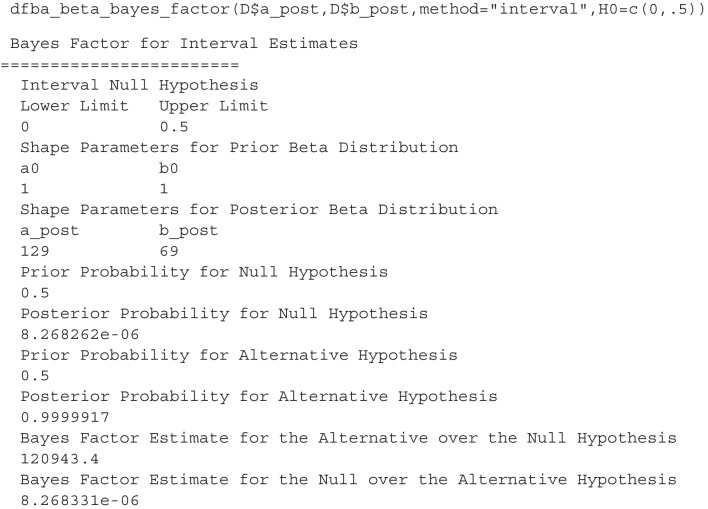



Please note that the Bayesian posterior point and interval estimates are for the $\phi _c$ concordance probability rather than for the $\tau _A$ correlation coefficient. However, because $\tau _A=2\phi _{c}-1$, the Bayesian posterior median for $\tau _A$ can be computed to be .3040526, which is close to the frequentist point estimate for $\tau _A$ that is shown above. Also, the limits for the posterior equal-tail 95% interval limits for $\tau _A$ can be computed in a similar fashion from the corresponding $\phi _c$ limits shown above to produce the [.167892, .4323704] interval estimate.

**Fig. 10:**
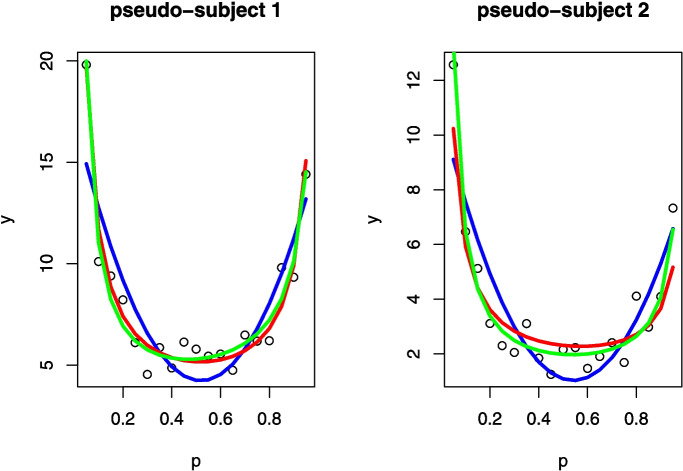
Examples of three models for fitting the points. Model M1 is the *blue line* based on Eq. 19. Model M2 is the *red line* from Eq. 20, and model M3 is the *green line* from Eq. 21

#### Distribution-free goodness-of-fit of a scientific model

The *dfba_bivariate_concordance* function can also be used in goodness-of-fit (GOF) testing that compares the observed data of a univariate continuous measure with the corresponding theory-based estimates from a scientific model that are based on a function of some *known* independent variable(s). There also might be several alternative scientific models that arise from different theoretical perspectives. Ultimately, for any of the theories, there will be a model-based estimate $\hat{y}_{M}$ for each observed value $y_{obs}$. That is, after fitting the theory to the observed data, a given model (denoted as *Mx*) will result in a collection of pairs $(y_{obs_i},\,\hat{y}_{Mx_i})$, $i=1,\cdots ,N$. If the model function is perfectly correct, and if there are trivially small measurement errors relative to the magnitude of the change in *y* between conditions, then there should be a correlation of 1 between the observed scores for various conditions and the fitted model estimates for those conditions. However, in reality, there are measurement errors, and the model may be incorrect. There are a number of frequentist GOF measures that are possible (e.g., Cramér, 1928; Massey, 1951), but these measures are not robust to the effect of outliers, and they make debatable parametric assumptions about the distribution of the fit residuals.

It is a challenging problem to ascertain which scientific theory is the best fit because it is possible that an incorrect model, which has many fitting parameters, can appear to be a good fit to the data. Consequently, in evaluating the scientific model it is important to penalize a model based on the number of free-fitting parameters. Thus, the problem of assessing the fit to data of a theoretical model is a complex topic, and it is beyond the scope of this paper to fully address. A detailed discussion of the GOF issue can be found in Chechile and Barch (2022). Chechile and Barch (2022) also showed that the concordance proportion between the observed scores $y_{obs}$ and the corresponding model-based estimates $\hat{y}_{M}$ could be a sensitive metric for discriminating among rival theories. It is also a robust measure with respect to the problem of outlier scores, and it does not require making parametric statistical assumptions about the unknown measurement error. It is also noteworthy that the concordance GOF assessment method is agnostic as to the method for obtaining a fit of the model parameters (i.e., the procedure for finding the $\hat{y}_{M}$ values). Mathematical models in the behavioral sciences typically have free-fitting parameters that have to be estimated before the model is fully specified. There are many ways that the model parameters can be estimated. For example, the point estimates for the model parameters could be based on maximum likelihood estimation; or the fit could be based on a least-squares error fit from a search of the model parameter space; or the fit might be based on the means of the posterior marginal distributions for the parameters. Any parameter fitting method will suffice for the goodness-of-fit concordance analysis, although it is reasonable to expect an analyst to employ the same fitting method for each of the candidate theoretical models. What is being assessed with a GOF test is the mathematical form of the scientific model. The concordance GOF assessment differs from parametric tests in that it does not assume a statistical model for the error residuals.

As a behavioral science application, suppose that there is a psychological phenomenon whereby people produce numerical responses $y$ according to some nonlinear function of a known independent variable called $p$. In a hypothetical experiment, $y$ is measured for several individuals at 19 discrete values for $p$ that range from $0.05$ to $0.95$ in steps of $0.05$. The three candidate models (M1, M2, and M3) being evaluated are:

19 $$\begin{aligned} M1: y= &  c_0 + c_1p +c_2 p^2\,\end{aligned}$$

20 $$\begin{aligned} M2: y= &  \frac{sa(-\log (p))^{a-1}}{p}\,\end{aligned}$$

21 $$\begin{aligned} M3: y= &  \frac{s(1-p)^{b-1}}{p^{a}}\left( b-a+\frac{a}{p}\right) \, \end{aligned}$$

where $c_0$, $c_1$, and $c_2$ are free-fitting parameters per individual for model *M*1; whereas for model *M*2, there are two parameters per individual (i.e., the *s* and *a* parameters) and for model *M*3, there are three adjustment parameters per individual, which are *s*, *a*, and *b*.

Let us consider two hypothetical experimental participants that are labeled as pseudo-subject 1 and pseudo-subject 2 to stress the fact that the data are artificial. The “observed” scores are computer-simulated values based on a nonlinear model plus an unbiased Gaussian error. The parameters of the true data-generated model differ for the two pseudo-subjects, and the standard deviation of the unbiased Gaussian error differ between the two pseudo-subjects. Figure 10 shows the “observed” values of the dependent variable $y$ along with lines of best fit for each of the three models. Importantly, it is possible that none of these three models is correct.

Because there are three fitting parameters for each pseudo-subject for model M1, there are a total of six fitting parameters used to obtain the model-based estimates. Model M2 has a total of four fitting parameters, and model M3 has a total of six fitting parameters. In general, the quality of any fit increases with the number of fitting parameters, so it is necessary to make an adjustment to the fit quality based on the number of parameters. There are adjustments for GOF statistics that employ a parametric likelihood function. The Akaike information criterion (AIC) (Akaike, 1973) and the Bayesian information criterion (BIC) (Schwarz, 1978) are examples of penalized metrics that are based on a parametric likelihood function. These methods depend on the validity of the parametric error model for the fit residuals. The $\phi _c$ concordance parameter is a distribution-free measure of association, but it still needs to be adjusted to take in account the number of free-fitting parameters. Chechile and Barch (2022) reduced the number of concordance values from $n_c$ to take into account the effect of having free-fitting parameters in the scientific model. In the Chechile–Barch approach there are $\frac{N (N-1)}{2}$ comparisons that are not forced when there are no fitting parameters. It is assumed that each fitting parameter effectively causes one condition to be lost for any comparisons to the other conditions. So, with *m* fitting parameters there are effectively *m* lost conditions leaving $N-m$ conditions that can be compared. Consequently, with *m* fitting parameters, there are $\frac{(N-m)(N-m-1)}{2}$ comparisons that are not forced. Thus, the number of lost comparisons by having *m* fitting parameters is $Lc=\frac{N(N-1)}{2}-\frac{(N-m)(N-m-1)}{2}$. Upon algebraic simplification, it follows that $Lc=Nm-\frac{m(m+1)}{2}$. Hence, the Chechile–Barch adjustment for *m* fitting parameters is to subtract *Lc* from *nc*. Thus, the adjusted number of concordant differences is $n_{c}^{*}=n_c-Nm+\frac{m(m+1)}{2}$. The number of discordant differences is not adjusted (i.e., $n_{d}^{*}=n_d$). Chechile and Barch (2022) defined an adjusted concordance parameter as $\phi _{c}^{*}=\frac{n_{c}^{*}}{n_{c}^{*}+n_d}$. The posterior distribution for the $\phi _{c}^{*}$ parameter is a beta distribution with shape parameters $a=n_{c}^{*}+a_{0}$ and $b=n_{d}+b_0$ where $a_0$ and $b_0$ are shape parameters for the prior. The *dfba_bivariate_concordance()* function implements this adjustment whenever the user provides a value for the *fitting.parameters* argument.

To implement the *dfba_bivariate_concordance()* function to do a distribution-free Bayesian analysis of the GOF for the three models, we first can create a vector called *yobs* for the 38 observed scores. Three other vectors are also created for the corresponding 38 theoretical estimates for each of the models. These vectors of theoretical values are called *yM1*, *yM2*, and *yM3*. Given these four vectors, the following function calls would evaluate the three models in terms of the $\phi _{c}^{*}$ statistic.

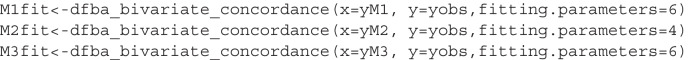



Table 6 has the key statistics for the Bayesian distribution-free assessment for the three models. Since the posterior distribution for $\phi _{c}^{*}$ is a beta distribution with known shape coefficients, the 95% highest density interval (HDI) can be found by using the *dfba_beta_descriptive()* function. For example, for model M1 the following function call can be used to find the HDI interval:

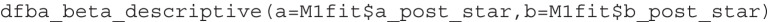



From *dfba_bivariate_concordance()* function calls, values for $n_c$, $n_d$, and $\phi _c$, $n^{*}_c$ are found for each model, and from the follow-up *dfba_beta_descriptive()* function calls, the HDI for $\phi ^{*}_c$ is found for each of the models. Please note that the best model in terms of the median value for $\phi _c^{*}$ is M3.

**Table 6:** The Bayesian distribution-free goodness-of-fit statistics for the three models from Eqs. 19-21 are provided along with 95% highest-density (HDI) intervals for the $\phi _{c}^{*}$

Model	$n_c$	$n_d$	$\phi _c$	$n_{c}^{*}$	$\phi _{c}^{*}$	HDI $\phi _{c}^{*}$
M1	616	87	.876	409	.824	[.790, .856]
M2	623	80	.885	481	.857	[.827, .885]
M3	639	64	.908	432	.870	[.840, .899]

Since the Bayesian $\phi ^{*}_c$ parameter has a posterior beta distribution, so it is straightforward to statistically assess the difference between rival models. To see how distribution-free model comparisons can be made, let us show with the following R code how the *dfba_beta_contrast()* function can be used to compute the probability of a difference between two models as well as compute a Bayes factor for the difference between models.

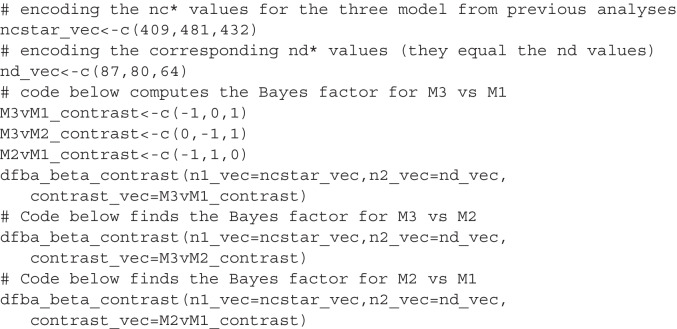



The Bayes factor $BF_{10}$ between models M3 and M1 is greater than 40, so, model M3 clearly is a better fit than model M1. However, the Bayes factor between models M3 and M2 is less than 3, so there is not a highly reliable difference in the fit quality after the adjustment for the number of fitting parameters. Model M2 is trending towards being better than model M1, but the Bayes factor for a difference is about 12, so it is not reliably better than M1.

### Bayesian analysis of rank-order contingency tables

Goodman and Kruskal (1954, 1959, 1963, 1972) introduced a gamma correlation for measuring the degree of bivariate association for an ordered-contingency table. The rows correspond to the rank of the *X* variate, whereas the columns correspond to the rank for the *Y* variate. The entry in the (*i*, *j*) cell is the frequency of bivariate observations where the *X* variate has a rank score of *i* while the *Y* variate has a rank score of *j*. The Goodman and Kruskal (1954) definition for $\gamma$ is as follows.

22 $$  \begin{aligned} \gamma= &  \frac{P(X[NONSPACE] \& Y\,\, agree\,\, in\,\, order)-P(X[NONSPACE] \& Y\,\,disagree\,\,in\,\,order)}{1-P(X[NONSPACE] \& Y\,\,tied)}. \end{aligned}$$

The *G* sample statistic is also computed as

23 $$\begin{aligned} G = \frac{N_s-N_d}{N_s+N_d}, \end{aligned}$$

where $N_s$ is the number of agreements between the orders of *X* and *Y*, and $N_d$ is the number of disagreements. Moreover

24 $$\begin{aligned} N_s= &  \sum _{i=1}^{R-1} \sum _{j=1}^{C-1} n_{ij} \left( \sum _{p=i+1}^{R} \sum _{q=j+1}^{C} n_{pq} \right) ,\end{aligned}$$

25 $$\begin{aligned} N_d= &  \sum _{i=1}^{R-1} \sum _{j=2}^{C} n_{ij} \left( \sum _{p=i+1}^{R} \sum _{q=1}^{j-1} n_{pq} \right) . \end{aligned}$$

where $n_{ij}$ is the frequency for the *i*th row and the *j*th column in the table and *R* and *C* are the respective total number of rows and columns (Siegel & Castellan, 1988). Chechile (2020b) showed that $n_c$ and $n_d$, as defined for the Kendall tau A, are respectively equal to the $N_s$ and $N_d$ values for the Goodman-Kruskal *G* statistic. Thus, it follows that the *G* sample statistic is equivalent to the sample $\tau _A$ from Eq. 14.

Given that $\tau _A$ along with its concordance parameter $\phi _c$ can be used to examine a rank-based contingency table, then why use the *dfba_gamma()* function rather than the previously discussed *dfba_bivariate_concordance()* function? The answer to this question is $\gamma$ can be more easily encoded as a table directly. For example, suppose researchers were interested in the following rank-order contingency table:

**Table:** 

	$R_{Y}=1$	$R_{Y}=2$	$R_{Y}=3$	$R_{Y}=4$	*total*
$R_{X}=1$	38	4	5	0	47
$R_{X}=2$	6	40	1	2	49
$R_{X}=3$	4	8	20	30	62
*total*	48	52	26	32	158

To do this problem with the *dfba_bivariate_concordance()* function would require creating *X* and *Y* paired variates where each vector has 158 values. However, the data can more easily be encoded as an R object that is either a matrix or a table. For example, the following coding shows how to encode the above hypothetical data as either a matrix called *N* or as a table called *T*.

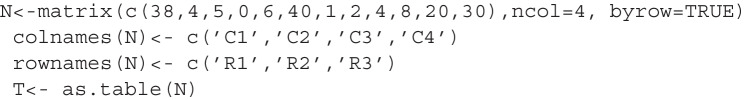



Thus, the *dfba_gamma()* function is a more convenient way to code the bivariate information when the data are already in the form of a rank-based contingency table.

The *dfba_gamma()* function has one required argument and three optional arguments. The required argument is call *x*, and it is set equal to a previously encoded R object that is an $I_{row} \times J_{col}$ rank-based contingency table or matrix. The three optional arguments are *a0*, *b0*, and *prob_interval*, and these arguments have the same meaning as the arguments with the same names in the *dfba_binomial()* function that was discussed in subsection “The dfba_binomial() function”. To implement a Bayesian concordance analysis for either the matrix or the table, the user simply needs to execute one of the following two instructions:

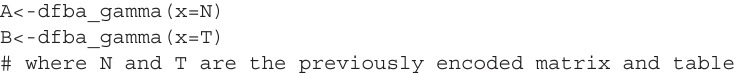



The *A* and *B* R objects are identical, and they contain the output information about the Goodman-Kruskal gamma as well as the population concordance parameter $\phi _c$. It is also easy to plot the prior and posterior for the $\phi _c$ parameter for the above problem with an instruction such as *plot(A)*.

## Summary and concluding remarks

The DFBA package brings together for the first time a set of distribution-free Bayesian analytic methods in software especially designed to benefit both seasoned data analysts and newcomers to Bayesian statistical inference. These methods include tools for analyzing observations from repeated measures and independent groups, analyzing bivariate concordance and contingency tables, assessing the goodness-of-fit of scientific function models, and conducting power analyses. The current paper serves as a technical guide to using the package and as a primer for these recently developed methods.

It is argued in this paper that the Bayesian inferential framework is better suited to the goals of researchers than orthodox frequentist statistics because unknown population parameters in the Bayesian approach are allowed to have a probability representation. Unlike the frequentist approach, Bayesian statistics provide for a probability representation for the unknown population parameters. Yet most Bayesian parametric analyses assume a model of Gaussian statistical error. Although parametric Bayesian analyses can be useful, a careful researcher should be concerned that the data might not satisfy the parametric assumptions. The functions in the DFBA package can be used as an alternative statistical analysis that does not depend on the data being sampled from a normal distribution with the same variance in each condition. Furthermore, there are many applications where the measured quantities are not continuous (i.e., categorical or ranked data). Again, the DFBA package can be used for those types of studies. Thus, the DFBA package of R functions has three major strengths: (1) it is Bayesian, (2) it does not depend on any particular distribution for measurement error, and (3) for continuous measures, it only uses the rank-order information, so it is insensitive to the influence of extreme scores or outliers. It is also open-source software that can be downloaded from CRAN, and used by anyone without cost.

Nonetheless, one might worry that when the measured quantity is continuous that there is a major loss of power relative to a parametric test. However, the power analysis functions in the DFBA package show that the loss of power is trivially small when the data are in fact are from a normal distribution. However, when the data are distributed as any of eight alternative models, which are available in the DFBA package, the distribution-free Bayesian methods actually have greater power! See Fig. 9 to examine the power curves for data from a normal distribution along with eight alternative models for statistical error. This finding about the relative power for the nonparametric methods underscores the fact that there is an enormous amount of information carried by only the rank-order of the scores. So, the DFBA package of functions is a powerful, yet robust, tool for researchers to employ. Moreover, the DFBA function calls are easy to execute, and the package has extensive online documentation plus vignettes to assist the user.

## Data Availability

Not applicable

## Appendix A Bayes factor tutorial

If $BF=\max (BF10, BF01)$, then the question is how large should *BF* be in order to be evidence in favor of a hypothesis? There is no set answer to this question, but several proposals have been made. The original suggestion (Jeffreys, 1961) was that a *BF* greater than 10 was *strong*, and a *BF* greater than 100 was *decisive*. Kass and Raftery (1995) instead proposed more stringent values for those two verbal adjectives; they suggested that a *BF* greater than 20 was *strong* and a *BF* value greater than 150 was *decisive*. Lee and Wagenmakers (2014) described a Bayes factor in the range of 3 to 10 as *moderate evidence* and characterized a Bayes factor in the 10 to 30 range as *strong evidence*.

Chechile (2020b) considered the Bayes factor from a formal expected-utility decision framework where there are *three* possible decision actions: (1) *deciding in favor of* $H_0$, (2) *deciding in favor of* $H_1$, and (3) *deciding not to make a decision yet*. In this analysis, there is a positive value utility $U_c$ for the reward of making a correct decision, and there is disutility (or scientific regret) of $-U_w$ for making a wrong decision, where $U_w$ is a positive number, so $-U_w$ is negative. When there is no decision made, then the scientist is not wrong by making a mistaken claim, but this outcome lacks the same reward as making a correct decision. For this case of not making a scientific claim, there is a positive utility of $rU_c$, where *r* is a value on the $(0,\,1)$ interval. Yet both extremes for *r* on this interval seem unrealistic. Consequently, (Chechile, 2020b) assumed a midpoint value by setting $r=0.5$. The following equations are the resulting expected utilities for the three decision alternatives:

$$\begin{aligned} E(U(H_0))= &  U_c\,P(H_0\,|\,D) - U_w\,P(H_1\,|\,D),\\ E(U(H_1))= &  -U_w\,P(H_0\,|\,D) + U_c\,P(H_1\,|\,D),\\ E(U_{no\,\,decision})= &  \frac{1}{2} U_c. \end{aligned}$$

The recommended action is to select the decision alternative with the maximum expected utility. Given this utility framework and based on the assumption that the priors for the two hypotheses are equal (i.e., $P(H_0)=P(H_1)$), Chechile (2020b) proved a theorem that showed that to decide on either $H_0$ or $H_1$, the Bayes factor $BF=max(BF10,\,BF01)$ needed to be larger than the quantity $\frac{2\,U_w}{U_c}+1$.^27^ In essence, the major competition for making a decision for either the null or the alternative hypothesis is the status quo of not making a decision at all because *BF* is not large enough. For example, if the regret disutility for making an erroneous decision is nine times larger than the reward utility for a correct decision, then that would mean the scientist should use a Bayes factor threshold criterion of 19 for making claims. It is assumed that most scientific fields prefer setting $U_w$ much greater than $U_c$ because there usually is a conservative bias for not making mistaken claims. Chechile (2020b) showed that if the ratio $\frac{U_w}{U_c}$ is in the range of $[4,\,9)$ corresponds to a Bayes factor threshold of $[9,\,19)$, which is characterized as “a promising but risky bet”. If $\frac{U_w}{U_c}$ is in the range of $[9,\,49)$ corresponds to a Bayes factor threshold of $[19,\,99)$, which is characterized as “a good bet – too good to disregard”. If $\frac{U_w}{U_c}$ is in the range of $[49,\,100)$ corresponds to a Bayes factor of $[99,\,201)$, which was characterized as a “strong bet – irresponsible to avoid”. Similarly (Chechile, 2020b) used the adjectival phrases of: “a very strong bet” for a *BF* in the $[201,\,2001)$ range, “nearing certainty” for a *BF* in the $[2001,\,20,001]$ range, and “virtually certain” when $BF>20,001$.

If the third decision option of *not deciding* were omitted from the utility analysis, then the decision between $H_0$ and $H_1$ would be strictly based on the posterior odds. Effectively without the third decision option of *not deciding* there would be a Bayes factor threshold of 1. Clearly, this threshold is not a scientifically credible position for hypothesis testing. Other theorists (e.g., Jeffreys, 1961; Kass and Raftery, 1995) set higher thresholds than 1 for the Bayes factor of scientific claims, but they did not provide a formal expected-utility analysis to justified their suggestions. Nonetheless, their recommendations are implicitly consistent with the status quo option of *no decision* being the primary rival to either of the other two decision alternatives.

The researcher can employ their own criteria for interpreting a Bayes factor value. More realistically, however, the decision criteria for hypothesis testing is an issue that various scientific disciplines and journals need to set. From the Chechile (2020b) framework, the criteria is effectively the scientific field collectively making a choice about the ratio of $U_w$ to $U_c$. For example, in particle physics, there is currently a five-sigma standard for the acceptance of a new finding (Franklin, 2013). While this criterion is expressed in terms of a frequentist *p* value, the Bayesian counterpart to the five-sigma criterion would be Bayes factor $BF\ge 3.5 \times 10^{6}$. However, this criterion is higher than other fields in the physical science, such as astronomy where a three-sigma criterion is more typical. A three-sigma criterion corresponds to a Bayes factor criterion of $BF\ge 740$. These very high criteria may be too large for the behavioral sciences where the frequentist $p\le .05$ has been widely used; this criterion is effectively equal to a $BF\ge 19$ standard. Yet (Chechile, 2020b) stressed that from a Bayesian decision-making framework there is always a chance, however small, that the scientific decision is incorrect. For this reason, the *dfba_beta_bayes_factor()* function provides the prior and posterior probabilities for both hypotheses in addition to the Bayes factor.

For the above Bayes factors discussion, both the null and alternative are interval hypotheses, but some investigators want the null hypothesis to be a single point (e.g., $H_0:\,\phi =\phi ^{*}$). Yet all point hypotheses have a probability measure of zero because for a continuous random variable a mathematical point cannot have a nonzero probability. Nonetheless, there is still a Bayes factor for a point-null hypothesis, but it is computed differently. To see how it is computed, let us rearrange the terms in the definition of the Bayes factor in the following fashion:

26 $$\begin{aligned} BF10 = [p(H_1|D)/p(H_1)] \cdot [p(H_0)/p(H_0|D)]. \end{aligned}$$

The first term in Eq. 26 is $1/1 = 1$ because the alternative hypothesis of $H_1:\,\phi \ne \phi ^{*}$ is 1 for both the prior and the posterior. The second term in Eq. 26 appears to be undefined because it is of the form of 0/0. However, by using L’Hôspital’s rule, this term is the ratio of the prior probability density at the null point divided by the posterior probability density. It thus follows that $BF10=f(\phi ^{*})/f(\phi ^{*}|D)$. If the posterior probability density at the null hypothesis point is less than the prior probability density, then $BF10>1$. This method for finding the Bayes factor is called the Savage–Dickey method (Dickey & Lientz, 1970). It should be pointed out that even if the Bayes factor *BF*01 is very large, the posterior probability for a point-null hypothesis is still zero, because all mathematical points only possess probability density rather than probability. Thus, the Savage–Dickey Bayes factor makes little sense for trying to build evidence for the alternative hypothesis because the alternative hypothesis has a probability of 1 for both the prior and the posterior. While the wisdom of using the Savage–Dickey approach is questionable, the DFBA package will allow for Bayes factors for both interval-null hypotheses as well as for point-null hypotheses.

If the Savage–Dickey Bayes factor test results in a large value for *BF*01, then it is also likely that the probability interval estimate will include the value of the null point $\phi ^{*}$. So, investigators, who are interested in a specific mathematical point, can always find a posterior interval estimate for the parameter and examine if the null point is inside the interval.

Even when the Bayes factor is below the user’s criterion for making a scientific claim, there is still some descriptive information value about the relative strength of evidence between the two hypotheses of interest. Also, from a Bayesian perspective, information from one study can be incorporated into the prior for the analysis of another study provided that the research protocols are matched and the hypotheses are the same. In this case, the combined Bayes factor from two separate studies is the product of the two Bayes factors.^28^ For example, if the Bayes factors for two separate studies were respectively $BF10(1)=5.7$ and $BF10(2)=6.8$, then the combined Bayes factor is $BF10(combined)=BF10(1) \times BF10(2)=38.76$, which exceeds the $BF\ge 19$ criterion. So, in this hypothetical example, two studies that fail to make a scientific claim can be combined to arrive at a decision. This idea generalizes to any number of separate studies that examine the same hypotheses and employ the same research protocols. Of course the combined Bayes factor can also downgrade a scientific claim as well. For example, if a study reports a Bayes factor of $BF10(1)=25.3$, but a follow-up replication study finds a $BF10(2)=.3125$, then the combined Bayes factor for this hypothetical case is $BF(combined)=7.906$, which is now below a Bayes factor criterion value of 19.

Finally, it should be acknowledged that the Bayes factor has some limitations. For example, (Gelman & Shalizi, 2013) has criticized the Bayes factor as a model comparison tool because it is a distortion of the process that scientists are faced with in trying to understand data. These authors point out that the Bayes factor is a comparison between two models, but the data analyst might have an unlimited number of possible candidate models for understanding the error structure of the data. Questions about the probability model for the errors and questions about the possible presence of mixtures are not limited to two mutually exclusive alternatives. While this point is valid, it is not particularly relevant for the applications of the Bayes factor in the DFBA package because (1) we are focused on distribution-free analyses (in part to avoid concerns about the problem of unknown measurement error and mixtures), and (2) the models examined with the Bayes factor in the DFBA package are mutually exclusive alternative hypotheses about a single population parameter. For this limited context, the Bayes factor is a useful tool for assessing the change in the posterior odds ratio for the two hypotheses relative to the prior odds ratio.

## Footnotes

1 The Barch and Chechile (2023) DFBA package is on the *Comprehensive R Archive Network* (CRAN) (R Core Team, 2024).

2 While there has been a growing awareness of the Bayesian approach in psychological science (e.g., Hoijtink and Chow, 2017a; Hoijtink and Chow, 2017b), the current official publication manual of the American Psychological Association (7th edition, 2020) still implicitly assumes that the user is doing a frequentist analysis, and it does not have any mention or acknowledgement of the Bayesian approach to statistical inference.

3 While the Clopper–Pearson confidence interval is consistent with frequentist theory, there are other intervals that are also called confidence intervals, but these methods violate the frequentist framework. Most notable is the Wilson CI and its approximation (Agresti & Coull, 1998; Wilson, 1927). These CIs are based on the assumption that the difference between the observed proportion $\frac{n1}{n}$ and population rate parameter $\phi$ is distributed as a normal distribution. Effectively, this approach lets the $\phi$ parameter have a distribution, which is inconsistent with the founding premise of the relative frequency theory. Chechile (2020b) showed that these CIs differed from the Clopper–Pearson CI, but they were reasonable approximations to the Bayesian interval estimate when $n \ge 50$. Users of this CI for the binomial rate parameter are unwittingly treating the parameter as a random variable, which is not allowed for a purely frequentist approach. Those users should instead be employing Bayesian estimation procedures.

4 Technically for a continuous random variable, the probability density at point *x*, which is denoted as *f*(*x*), is the derivative of the cumulative probability function at point *x*, which in turn is denoted as *F*(*x*), and it is computed as $\int _{L}^{x} f(r)\,dr$ where *L* is the lower limit for the support for the random variable.

5 Chechile (2020b) pointed out that some frequentist meta-analysis procedures make the *p* value error, which was previously discussed in subsection “Why do Bayesian analyses?”. He also noted that *If one wants to do a proper meta-analysis, then be Bayesian! *(p. 145)

6 This Bayes factor is usually denoted as $BF_{10}$, but in this paper the subscript is dropped to be consistent with DFBA documentation and output.

7 While *parametric Bayesian analyses* can often have cases where the posterior distribution does not have a closed form solution. For such cases, Markov chain Monte Carlo (MCMC) algorithms have been a valuable tool. MCMC procedures such as the Metropolis et al. (1953) algorithm or the Gibbs sampler (Geman & Geman, 1984), are approximate methods that have enabled a parametric Bayesian solution. MCMC methods are based on ergodicity theory (Birkhoff, 1931), and these techniques are approximate. There are issues with a MCMC about the starting position in the chain, the correlation among the samples, and the rate of convergence of the chain. Unlike a MCMC algorithm, the Monte Carlo sampling in all the DFBA functions are not based on a Markov chain. The random sampling does not require an initial guess. Also, unlike an MCMC algorithm, the Monte Carlo sampling used with DFBA functions does not need a burn-in period nor does it have an autocorrelation problem that must be mitigated. Rather the random sampling uses conventional Monte Carlo sampling procedures that are in the base R *stats* package. Thus, all the sampled points in the *dfba_beta_contrast()* function are valid independently sampled random scores from the posterior beta distributions for the separate conditions.

8 While orthogonal contrasts are easy to construct, there is no reason that the user’s research interest and the experimental design need to be limited to a set of orthogonal contrasts. For example, if the researcher has a single control condition and $K-1$ experimental treatment conditions, then it is reasonable that the data analyst would be interested in a non-orthogonal set of $K-1$ contrasts where each experimental condition is compared to the control condition. From the three-alternative decision-making framework discussed in Appendix A, the analyst might only make a scientific claim for either the alternative or null hypothesis on a subset of the $K-1$ where $BF=max(BF10, BF01)$ is sufficiently large.

9 A frequentist sign test would have also rejected the null hypothesis because it would have resulted in $p<.02$. So, a careful frequentist would have also detected the problem with the parametric *t* test for these data.

10 The choice of 200 intervals is arbitrary, but it is a reasonable value given the tradeoff between the smoothness of the posterior distribution and the computing time cost. A smaller value would run faster, but result in a more choppy discrete posterior distribution. A larger value would achieve only slightly more smoothness but would require a substantial increase in computational time.

11 It is possible in rare occasions for the sample $T^{+}$ to be a non-integer value when more that one block has the same |*d*| value. Yet for the small-*n* approach, the algorithm for sampling signed ranks will not produce a non-integer $T^{+}$. Consequently, in the small-*n* approach, the sample $T^{+}$ is rounded to be an integer to avoid this difficulty.

12 The reader should not confuse the $U_E$ and $U_C$ metrics with the $T_E$ and $T_C$ statistics that Wilcoxon (1945) employed when there are two independent variates. These *T* metrics sum the rank scores for each condition. These statistics are typically called the Wilcoxon–Mann–Whitney *T* statistics (Lehmann, 1975; Siegel & Castellan, 1988). The *T* statistics in general are different from the more minimalist *U* metrics, but when the sample sizes are equal and there are no ties, the *T* statistics are perfectly correlated with the *U* statistics.

13 For example, the *dfba_mann_whitney()* has a default value of 30,000 random samples per value for $\Omega _{Ei}=.0025+(i-1)(.005)$ for $i=1,\cdots , 200$.

14 The harmonic mean is $\frac{2n_E\cdot n_C}{n_E+n_C}$

15 The above analyses forced the large-sample approach to save time for readers who might choose to implement this example on their computers. However, without using the method=“large” option, the same conclusions as reported above would also be reached.

16 Please note that both frequentist power and Bayesian power are estimated as proportions over repeated random samples. A critic might be troubled by a Bayesian analysis that employed the concept of relative frequency as opposed to subjective probability. However, as Chechile (2020b) stressed, both relative frequencies and subjective probabilities can satisfy the Kolmogorov axioms for probability. From this perspective, there is no reason to avoid having a subjective probability for a population parameter, which is a fixed unknown quantity, and there is also no reason to avoid having a probability for the proportion over random data sets for finding a given statistical outcome when the data sets were randomly generated from probability models for the data for a hypothetical study. The first use of probability is what is done for the Bayesian analysis of an actual experiment, and the second use of probability is the estimation for finding an effect from a Bayesian analysis based on the research plan.

17 Readers, who wish to learn more about the probability models and the applications associated with the models, can find documentation in the vignette for the *dfba_sim_data()* function.

18 However, R software developers might be interested in using the *dfba_sim_data()* function for some of its useful features. The R documentation and the vignette about the *dfba_sim_data()* function are sufficiently informative so that R developers can use the function as a tool for the building of other functions.

19 Although the user is free to change the value of *n_min*, the function will not allow the user to enter a value less than 20. The reason for this constraint is that the Bayesian analyses use the large-sample approximation method to speed up execution time. These analyses will be reasonably accurate when the sample size is at least 20.

20 For all the probability models the variate has a shape parameter. For example, for the normal model the two shape parameters arguments are the standard deviations for the respective first and second conditions. Both the R documentation and the vignette on the power functions for the DFBA package provide information about the shape parameters for all nine probability models.

21 When the scale factor for a logistic distribution is 1, the standard deviation is equal to $\frac{\pi }{\sqrt{3}}$, but if the scale factor is $\frac{\sqrt{3}}{\pi }$, which is approximately .551, then the logistic distribution has a standard deviation of 1. Consequently, the logistic distribution is an excellent approximation to the standard normal distribution when its scale factor is .551.

22 The user can enter any positive integer equal or greater than 20 for the *n* argument. This restriction on *n* is required because the software uses the large-*n* approximation for the Bayesian analyses.

23 What is commonly known as the Kendall correlation was initially proposed by Fechner (1897), but independently rediscovered in the Kendall (1938) paper. The history of rank-based correlations is explored in the Kruskal (1958) paper.

24 The ranks for the ten points in this example are: (1, 2.5, 2.5, 4.5, 4.5, 6.5, 6.5, 8, 9.5, 9.5) for the first measure, whereas the corresponding ranks for the second measure are: (1, 2, 4.5, 4.5, 4.5, 4.5, 8, 8, 8, 10). Thus, $T_X=\frac{2\cdot 1}{2}+\frac{2\cdot 1}{2}+\frac{2\cdot 1}{2}+\frac{2\cdot 1}{2}=4$, $T_Y=\frac{4\cdot 3}{2}+\frac{3\cdot 2}{2}=9$, and $T_{XY}= \frac{2\cdot 1}{2}$=1. From Eq. 15 it follows that $n_c+n_d=45-4-9+1=33$.

25 The frequentist analysis, however, sets the $\phi _c$ parameter to a fixed null value (e.g., $\phi _c$=.5), and based on this assumed null-hypothesis, the likelihood for getting $n_c$ *or more* concordant comparisons is calculated. Thus, the likelihood for the frequentist analysis concerns the likelihood for the observed data plus *some unobserved outcomes that are more extreme* for strictly the null hypothesis. However, upon completion of a study, a Bayesian analysis assesses the likelihood for the observed outcome, but that likelihood is computed for all possible values for the $\phi _c$ parameter. As discussed previously, the principle of only computing the likelihood of the observed data is called the *likelihood principle* (Barnard et al., 1962; Berger & Wolpert, 1988).

26 Chechile (2020b) described the classification of concordant and discordant comparisons as a censored Bernoulli process because the number of possible arrangements of comparisons ($k_{nc}$) where there are $n_c$ concordant comparisons and $n_d$ discordant comparisons must satisfy the transitivity of order. However, $k_{nc}$ does not matter because it appears in both the numerator and denominator of Bayes theorem, so it cancels out.

## Declarations

### Code Availability

All the functions in the DFBA package are available on the R CRAN repository.
